# Supplementary material for: ARHGEF26 enhances Salmonella invasion and inflammation in cells and mice
Source: PLoS Pathog. 2021 Jul 9;17(7):e1009713. doi: 10.1371/journal.ppat.1009713 (PMC8294491; doi:10.1371/journal.ppat.1009713)
Supplement: S1 Table — A: Genes used for stratification. B: Bacterial strains used in this study. C: Plasmids used in this study. D: Oligonucleotides used in this study. (DOCX) [file ppat.1009713.s001.docx]

S1 Tables

| Table A: Genes used for stratification. | | |
| --- | --- | --- |
| Gene | **Complex** | **Justification** |
| *ABI1* | WAVE | (1-3) |
| *ABI2* | WAVE | (1-3) |
| *ABI3* | WAVE | (1-3) |
| *ACTB* |  | (4-7) |
| *ACTR2* | ARP2/3 | (1, 2, 8) |
| *ACTR3* | ARP2/3 | (1, 2, 8) |
| *ARF1* |  | (3) |
| *ARF6* |  | (3, 9) |
| *ARHGEF26* (*SGEF*) |  | (10) |
| *ARPC1A* | ARP2/3 | (1, 2, 8) |
| *ARPC1B* | ARP2/3 | (1, 2, 8) |
| *ARPC2* | ARP2/3 | (1, 2, 8) |
| *ARPC3* | ARP2/3 | (1, 2, 8) |
| *ARPC4* | ARP2/3 | (1, 2, 8) |
| *ARPC5* | ARP2/3 | (1, 2, 8) |
| *BRK1* | WAVE | (1-3) |
| *CDC42* |  | (2, 8, 10, 11) |
| *CYFIP1* | WAVE | (1-3) |
| *CYFIP2* | WAVE | (1-3) |
| *CYTH2* (*ARNO*) |  | (3, 9) |
| *NCKAP1* | WAVE | (1-3) |
| *RAC1* |  | (2, 10-12) |
| *RHOG* |  | (10, 13) |
| *WASF1* | WAVE | (1-3) |
| *WASF2* | WAVE | (1-3) |

| **Table B: Bacterial strains used in this study** | | | | |
| --- | --- | --- | --- | --- |
| **Designation** | **Serovar** | **Genotype** | **Plasmid** | **Resistance** |
| CS093 | S. Typhimurium | Wild-Type (14028s) |  |  |
| DCK22 | S. Typhimurium | Wild-Type | p67GFP3.1 | Ampicillin |
| DCK483 | S. Typhimurium | Wild-Type | pWSK29 | Ampicillin |
| DCK484 | S. Typhimurium | Wild-Type | pWSK129 | Kanamycin |
| DCK89 | S. Typhimurium | ∆SopE2::tetR |  | Tetracycline |
| DCK95 | S. Typhimurium | ∆SopE2::tetR | p67GFP3.1 | Tetracycline, Ampicillin |
| DCK103 | S. Typhimurium | ∆sopB |  |  |
| DCK768 | S. Typhimurium | ∆sopB | pWSK29 | Ampicillin |
| DCK783 | S. Typhimurium | ∆sopB | pWSK129 | Kanamycin |
| DCK971 | S. Typhimurium | ∆prgH | pWSK129 | Kanamycin |
| DCK1062 | S. Typhimurium | ∆sopB∆sopE2 | p67GFP3.1 | Ampicillin |
| DCK1063 | S. Typhimurium | ∆sopB∆sopE2 | pWSK129 | Kanamycin |
| CS092 | S. Typhi | Wild-Type (Ty2) |  |  |
| DCK33 | S. Typhi | Wild-Type (Ty2) | p67GFP3.1 | Ampicillin |
| DCK305 | S. Typhi | ∆sopB |  |  |
| DCK308 | S. Typhi | ∆sopB | p67GFP3.1 | Ampicillin |
| DCK306 | S. Typhi | ∆sopE |  |  |
| DCK309 | S. Typhi | ∆sopE | p67GFP3.1 | Ampicillin |
| DCK307 | S. Typhi | ∆prgH |  |  |
| DCK310 | S. Typhi | ∆prgH | p67GFP3.1 | Ampicillin |
| DCK326 | S*.* Typhi | ∆sopB∆sopE | p67GFP3.1 | Ampicillin |
| DCK1127 | S. Typhi | ∆sopB∆sopE | pACYC184, p67GFP3.1 | Ampicillin, Chloramphenicol |
| DCK1128 | S. Typhi | ∆sopB∆sopE | pM515 (pACYC184:SopB (STM)), p67GFP3.1 | Ampicillin, Chloramphenicol |
| DCK1129 | S. Typhi | ∆sopB∆sopE | pM149 (pACYC184:SopE2 (STM)), p67GFP3.1 | Ampicillin, Chloramphenicol |

| Table C: Plasmids used in this study | | |  |  |
| --- | --- | --- | --- | --- |
| Bacterial Stock | **Parental Plasmid** | **Insert** | **Resistance** | **Source** |
| DCK18 | p67GFP3.1 |  | Ampicillin | (14) |
| DCK482 | pWSK29 |  | Ampicillin | (15) |
| DCK827 | pWSK129 |  | Kanamycin | (15) |
| CS943 | pCP20 |  | Ampicillin | (16) |
| CS946 | pKD4 |  | Kanamycin | (16) |
| HB2502 | pKD46 |  | Ampicillin | (16) |
| DCK718 | pCMV-Myc |  | Ampicillin | (17) |
| DCK719 | pCMV-Myc | ARHGEF26 | Ampicillin | (17) |
| DCK749 | pCMV-Myc | ARHGEF26 ∆ETNV (∆aa868-871) | Ampicillin | (18) |
| DCK750 | pCMV-Myc | ARHGEF26 aa1-400 | Ampicillin | (18) |
| DCK751 | pCMV-Myc | ARHGEF26 Catalytically Dead (E446A, N621A) | Ampicillin | (18) |
| DCK752 | pCMV-Myc | ARHGEF26 aa415-871 | Ampicillin | (18) |
| DCK753 | pCMV-Myc | ARHGEF26 PH-DH domain (aa431-792) | Ampicillin | (18) |
| DCK754 | pCMV-Myc | ARHGEF26 ∆SH3 (∆aa787-871) | Ampicillin | (18) |
| DCK757 | pCMV-Myc | ARHGEF26 ∆PH (∆aa656-726) | Ampicillin | This Study |
| DCK53 | pEGFP-C1 |  | Kanamycin | Clontech |
| DCK835 | pEGFP-C1 | FLAG-ARHGEF26 | Kanamycin | This Study |
| DCK837 | pEGFP-C1 | FLAG-ARHGEF26 Catalytically Dead (R446A, N621A) | Kanamycin | This Study |
| DCK839 | pEGFP-C1 | FLAG-ARHGEF26 ∆PH (∆∆aa656-726) | Kanamycin | This Study |
| DCK784 | pCS2 | RhoG | Ampicillin | This Study |
| DCK531 | pBVLuc |  | Ampicillin | (19) |
| DCK1013 | pBVLuc | ARHGEF26 rs993387 Locus Major Allele (HG02860) | Ampicillin | This Study |
| DCK1014 | pBVLuc | ARHGEF26 rs993387 Locus Minor Allele (HG02860) | Ampicillin | This Study |
| DCK534 | pRL-SV40P |  | Ampicillin | (19) |
| DCK1105 | pACYC184 |  | Chloramphenicol | (20) |
| DCK1106 (pM515) | pACYC184 | SopB (From *S.* Typhimurium) | Chloramphenicol | (21) |
| DCK1108 (pM149) | pACYC184 | SopE2 (From *S.* Typhimurium) | Chloramphenicol | (21) |

| Table D: Oligonucleotides | | |  |
| --- | --- | --- | --- |
| *Taqman Assays* | | | |
| Target Gene | **Assay ID** | **Source** |  |
| *ARHGEF26* | Hs00248943_m1 | ThermoFisher |  |
| *SCRIB* | Hs00363005_m1 | ThermoFisher |  |
| *DLG1* | Hs00938204_m1 | ThermoFisher |  |
| *RHOG* | Hs00750922_s1 | ThermoFisher |  |
| *RNA45S5* | Hs03928990_g1 | ThermoFisher |  |
|  |  |  |  |
| *Site Directed Mutagenesis* | |  |  |
| Target Gene | **Mutation** | **Forward** | **Reverse** |
| *ARHGEF26* | ∆PH domain (∆aa656-726) | CTCTTCCCGGTGGGGGAAGCCGCCTG | CAGGCGGCTTCCCCCACCGGGAAGAG |
|  |  |  |  |
| *Cut and Paste Cloning* | |  |  |
| Target Gene | **Source/Destination** | **Forward** | **Reverse** |
| *ARHGEF26* Constructs | pCMV Myc Constructs --> pEGFP3.1 | ATCGATCGATGTCGACGACTACAAGGACGACGATGACAAGATGGACGGCGAGAGCGAGGT | TTAAGCGCTATAGGATCCCTACACGTTGGTCTCCAGTC |
|  |  |  |  |
| *Lambda-Red Recombination* | |  |  |
| Target Serovar | **Target Gene** | **Forward for Cassette Generation** | **Reverse for Cassette Generation** |
| *S.* Typhimurium and *S.* Typhi | *sopB* | GAATGTTCCCACTCCCCTATTCAGGAATATTAAAAACGCTGTGTAGGCTGGAGCTGCTTC | ACGATTTAATAGACTTTCCATATAGTTACCTCAAGACTCACATATGAATATCCTCCTTAG |
| *S.* Typhimurium | *prgH* | CTGCTGCTATCGAGAACGACAGACATCGCTAACAGTATATGTGTAGGCTGGAGCTGCTTC | AAGGTGTTGCCATAATGACTTCCTTATTTACGTTAAATTACATATGAATATCCTCCTTAG |
| *S.* Typhi | *sopE* | ATATATAAATGAGTTATGTACATATAAAAGGATCATTACCGTGTAGGCTGGAGCTGCTTC | AGGAAGAGGCTCCGCATATTTTTTGGTTTTTCTGTGTTCACATATGAATATCCTCCTTAG |
| *S.* Typhi | *prgH* | CTGCTGCTATCGAGAACGACAGATATCGCTAACAGTATATGTGTAGGCTGGAGCTGCTTC | AAGATGTTGGCATAATGACTTCCTTATTTGCGTTAAATTACATATGAATATCCTCCTTAG |
|  |  |  |  |
| *Strain Confirmation* | |  |  |
| Target Serovar | **Target Gene** | **Forward** | **Reverse** |
| *S.* Typhimurium and *S.* Typhi | *sopB* | CCTGGTGCATAAAAGTCACATCC | CGGATTCATTAAAATAAACCTGTA |
| *S.* Typhimurium *and S.* Typhi | *prgH* | AATCCCTGTGTCCTGTGCGG | TATCCAGCGCCTCTGTTACC |
| *S.* Typhi | *sopE* | CATCAATCAGATGGACATAGCATTTGC | GACGGTTTAGCTCCGGAGTTAG |

1. Criss AK, Casanova JE. Coordinate regulation of Salmonella enterica serovar Typhimurium invasion of epithelial cells by the Arp2/3 complex and Rho GTPases. Infect Immun. 2003;71(5):2885-91.

2. Unsworth KE, Way M, McNiven M, Machesky L, Holden DW. Analysis of the mechanisms of Salmonella-induced actin assembly during invasion of host cells and intracellular replication. Cell Microbiol. 2004;6(11):1041-55.

3. Humphreys D, Davidson A, Hume PJ, Koronakis V. Salmonella virulence effector SopE and Host GEF ARNO cooperate to recruit and activate WAVE to trigger bacterial invasion. Cell Host Microbe. 2012;11(2):129-39.

4. Lilic M, Galkin VE, Orlova A, VanLoock MS, Egelman EH, Stebbins CE. Salmonella SipA polymerizes actin by stapling filaments with nonglobular protein arms. Science. 2003;301(5641):1918-21.

5. Hayward RD, Koronakis V. Direct nucleation and bundling of actin by the SipC protein of invasive Salmonella. EMBO J. 1999;18(18):4926-34.

6. Zhou D, Mooseker MS, Galan JE. An invasion-associated Salmonella protein modulates the actin-bundling activity of plastin. Proc Natl Acad Sci U S A. 1999;96(18):10176-81.

7. Francis CL, Starnbach MN, Falkow S. Morphological and cytoskeletal changes in epithelial cells occur immediately upon interaction with Salmonella typhimurium grown under low-oxygen conditions. Mol Microbiol. 1992;6(21):3077-87.

8. Stender S, Friebel A, Linder S, Rohde M, Mirold S, Hardt WD. Identification of SopE2 from Salmonella typhimurium, a conserved guanine nucleotide exchange factor for Cdc42 of the host cell. Mol Microbiol. 2000;36(6):1206-21.

9. Humphreys D, Davidson AC, Hume PJ, Makin LE, Koronakis V. Arf6 coordinates actin assembly through the WAVE complex, a mechanism usurped by Salmonella to invade host cells. Proc Natl Acad Sci U S A. 2013;110(42):16880-5.

10. Patel JC, Galan JE. Differential activation and function of Rho GTPases during Salmonella-host cell interactions. J Cell Biol. 2006;175(3):453-63.

11. Chen LM, Hobbie S, Galan JE. Requirement of CDC42 for Salmonella-induced cytoskeletal and nuclear responses. Science. 1996;274(5295):2115-8.

12. Ablain J, Xu M, Rothschild H, Jordan RC, Mito JK, Daniels BH, et al. Human tumor genomics and zebrafish modeling identify SPRED1 loss as a driver of mucosal melanoma. Science. 2018;362(6418):1055-60.

13. Patel JC, Galan JE. Investigating the function of Rho family GTPases during Salmonella/host cell interactions. Methods Enzymol. 2008;439:145-58.

14. Pujol C, Bliska JB. The ability to replicate in macrophages is conserved between Yersinia pestis and Yersinia pseudotuberculosis. Infect Immun. 2003;71(10):5892-9.

15. Wang RF, Kushner SR. Construction of versatile low-copy-number vectors for cloning, sequencing and gene expression in Escherichia coli. Gene. 1991;100:195-9.

16. Datsenko KA, Wanner BL. One-step inactivation of chromosomal genes in Escherichia coli K-12 using PCR products. Proc Natl Acad Sci U S A. 2000;97(12):6640-5.

17. Ellerbroek SM, Wennerberg K, Arthur WT, Dunty JM, Bowman DR, DeMali KA, et al. SGEF, a RhoG guanine nucleotide exchange factor that stimulates macropinocytosis. Mol Biol Cell. 2004;15(7):3309-19.

18. Awadia S, Huq F, Arnold TR, Goicoechea SM, Sun YJ, Hou T, et al. SGEF forms a complex with Scribble and Dlg1 and regulates epithelial junctions and contractility. J Cell Biol. 2019;218(8):2699-725.

19. He TC, Chan TA, Vogelstein B, Kinzler KW. PPARdelta is an APC-regulated target of nonsteroidal anti-inflammatory drugs. Cell. 1999;99(3):335-45.

20. Chang AC, Cohen SN. Construction and characterization of amplifiable multicopy DNA cloning vehicles derived from the P15A cryptic miniplasmid. Journal of bacteriology. 1978;134(3):1141-56.

21. Mirold S, Ehrbar K, Weissmuller A, Prager R, Tschape H, Russmann H, et al. Salmonella host cell invasion emerged by acquisition of a mosaic of separate genetic elements, including Salmonella pathogenicity island 1 (SPI1), SPI5, and sopE2. Journal of bacteriology. 2001;183(7):2348-58.
