# Supplementary material for: ARHGEF26 enhances Salmonella invasion and inflammation in cells and mice
Source: PLoS Pathog. 2021 Jul 9;17(7):e1009713. doi: 10.1371/journal.ppat.1009713 (PMC8294491; doi:10.1371/journal.ppat.1009713)
Supplement: S1 Fig — (A) Stratified QQ plots of host cell infection reveal that, in addition to rs993387, multiple SNPs in ARHGEF26 (blue diamonds) associate with S. Typhi or S. Typhimurium infection in H2P2 at lower p-values than expected by chance. (B) Removing SNPs in ARHGEF26 from the QQ plot removes any deviation of SNPs in SPI-1 associated genes from p-values expected by chance. (C) rs993387 association was observed across all four populations studied in H2P2 (IBS, Iberians from Spain; GWD, Gambian from the Western Divisions of The Gambia; ESN, Esan in Nigeria; KHV, Kinh in Ho Chi Minh City, Vietnam). Each dot represents a single LCL line averaged across three independent experiments. Black bar represents the median. LCLs in each population follow the trend of TT < GT < GG, except in GWD and ESN for Typhi where only 1 or 2 GG individuals were assayed. (D) A luciferase reporter system was generated to assess whether the rs993387 locus has enhancer activity. A roughly 5kb region was cloned from a heterozygous individual (HG02860, GWD) into pBV-Luc upstream of a minimal promoter and the firefly luciferase gene. Performing a dual luciferase experiment in HeLa cells revealed enhanced luciferase expression with the rs993387 locus. Bars represent the relative firefly luciferase/renilla luciferase activity, with vector set to 1. P-Values generated from one-way ANOVA with Tukey’s multiple comparisons test on the log transformed values. (E) siRNA targeting ARHGEF26 results in reduced expression in LCLs (HG01697, IBS). (F) siRNA targeting ARHGEF26, DLG1, SCRIB, and RHOG results in reduced expression in HeLa cells. Lines in E and F represent median fold change. Fold change is calculated as 2-ΔΔCT using RNA18S5 as a housekeeping control gene. (DOCX) [file ppat.1009713.s002.docx]

**
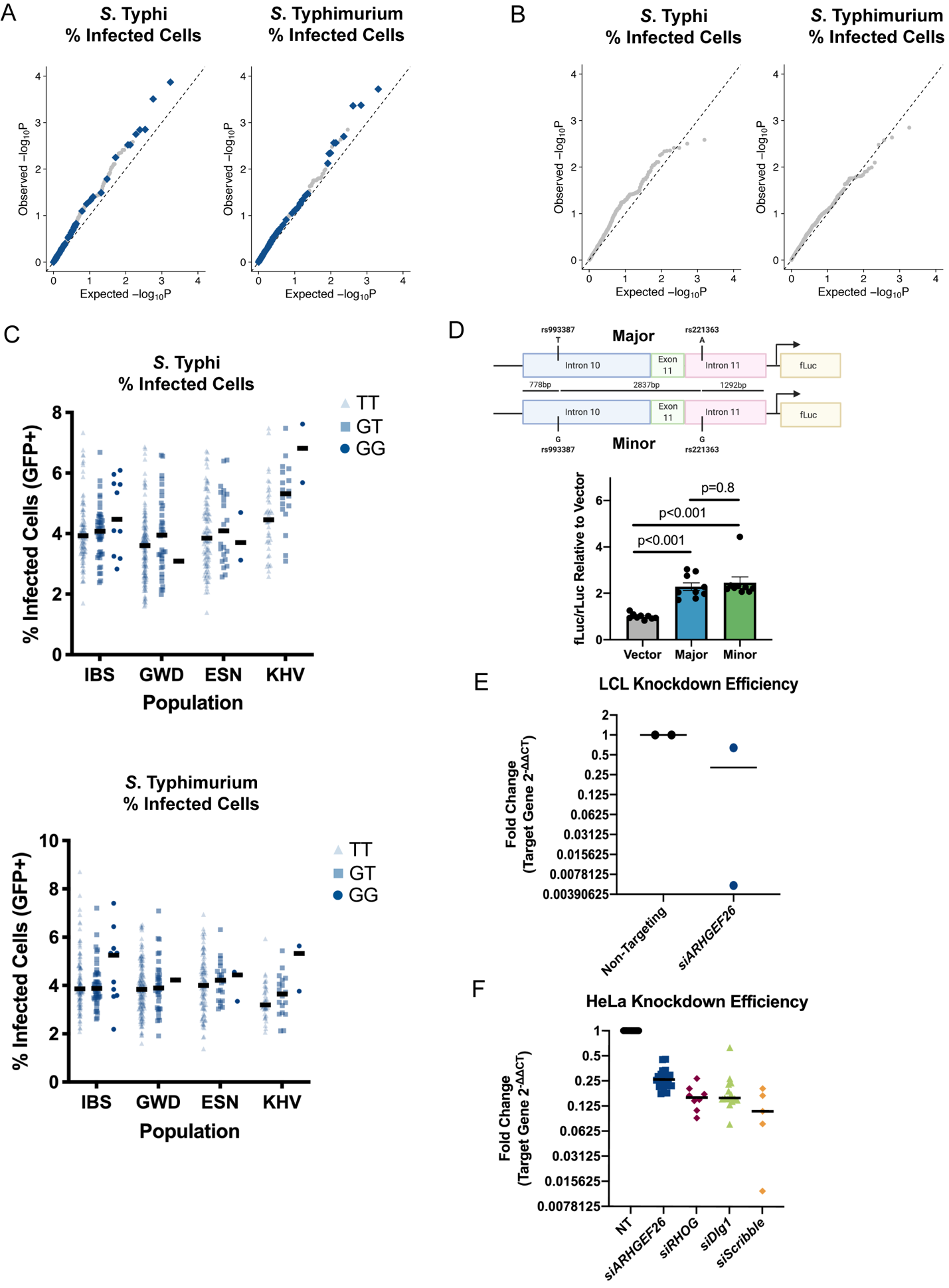
**

**S1 Fig: Natural variation in *ARHGEF26* and *ARHGEF26* knockdown associate with reduced *Salmonella* uptake**. (A) Stratified QQ plots of host cell infection reveal that, in addition to rs993387, multiple SNPs in *ARHGEF26* (blue diamonds) associate with *S.* Typhi or *S.* Typhimurium infection in H2P2 at lower p-values than expected by chance. (B) Removing SNPs in *ARHGEF26* from the QQ plot removes any deviation of SNPs in SPI-1 associated genes from p-values expected by chance. (C) rs993387 association was observed across all four populations studied in H2P2 (IBS, Iberians from Spain; GWD, Gambian from the Western Divisions of The Gambia; ESN, Esan in Nigeria; KHV, Kinh in Ho Chi Minh City, Vietnam). Each dot represents a single LCL line averaged across three independent experiments. Black bar represents the median. LCLs in each population follow the trend of TT < GT < GG, except in GWD and ESN for Typhi where only 1 or 2 GG individuals were assayed. (D) A luciferase reporter system was generated to assess whether the rs993387 locus has enhancer activity. A roughly 5kb region was cloned from a heterozygous individual (HG02860, GWD) into pBV-Luc upstream of a minimal promoter and the firefly luciferase gene. Performing a dual luciferase experiment in HeLa cells revealed enhanced luciferase expression with the rs993387 locus. Bars represent the relative firefly luciferase/renilla luciferase activity, with vector set to 1. P-Values generated from one-way ANOVA with Tukey's multiple comparisons test on the log transformed values. (E) siRNA targeting *ARHGEF26* results in reduced expression in LCLs (HG01697, IBS). (F) siRNA targeting *ARHGEF26, DLG1, SCRIB,* and *RHOG* results in reduced expression in HeLa cells. Lines in E and F represent median fold change. Fold change is calculated as 2^-∆∆CT^ using RNA18S5 as a housekeeping control gene.
