# Supplementary material for: ARHGEF26 enhances Salmonella invasion and inflammation in cells and mice
Source: PLoS Pathog. 2021 Jul 9;17(7):e1009713. doi: 10.1371/journal.ppat.1009713 (PMC8294491; doi:10.1371/journal.ppat.1009713)
Supplement: S2 Fig — ARHGEF26, RHOG, and the GFP-AKT-PH constructs were overexpressed in either HeLa or HEK293T cells. Cells were lysed, and protein extract was diluted in the listed blocking buffer before incubation on PIP strips. No robust ARHGEF26 signal on dotted phosphoinositide species could be detected following immunostaining. Key in bottom right displays location of each phosphoinositide species using a PIP strip incubated with GFP-AKT-PH (also shown in top left) as an example. (DOCX) [file ppat.1009713.s003.docx]

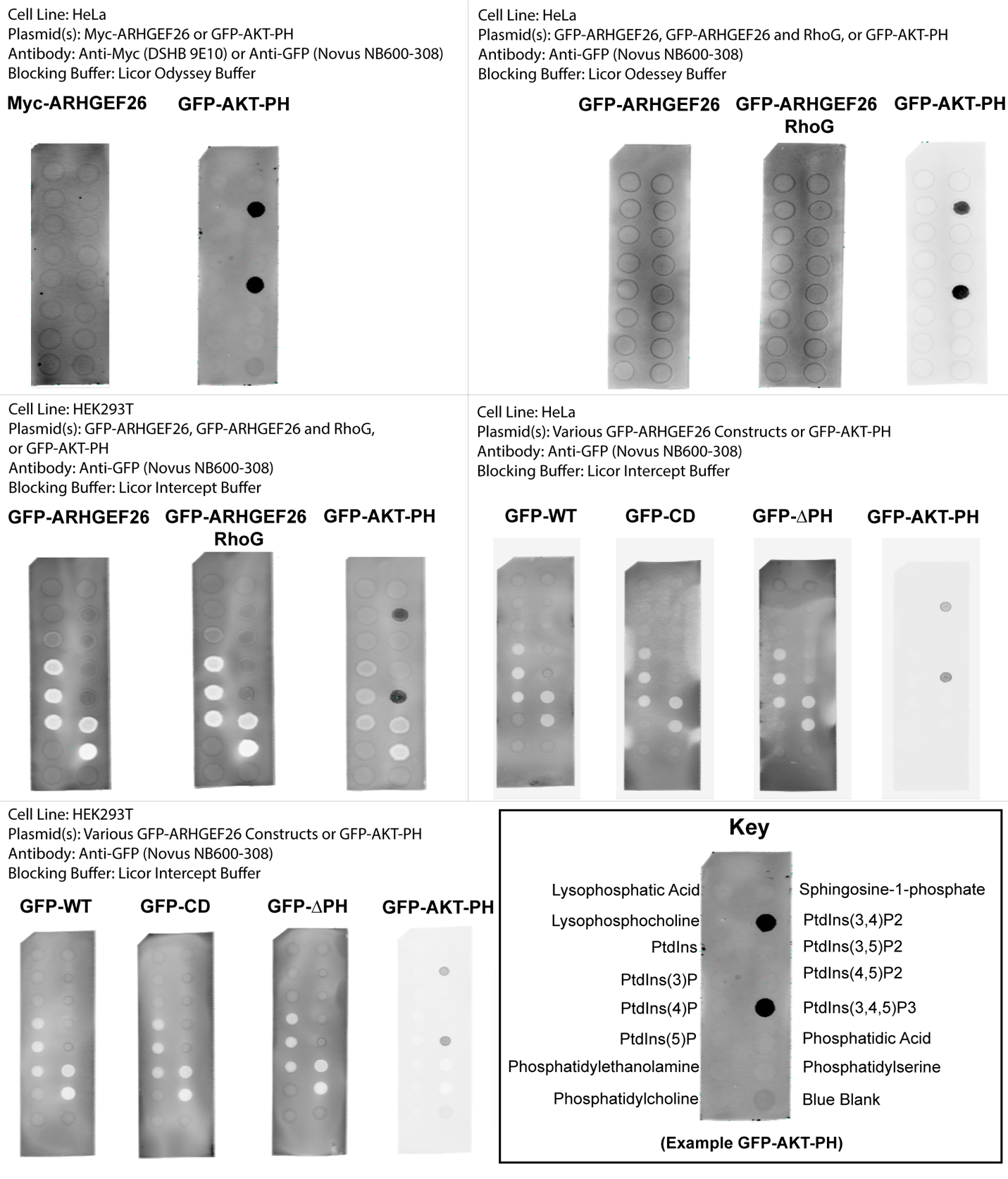


**S2 Fig: ARHGEF26 does not demonstrate phosphoinositide binding.** ARHGEF26, RHOG, and the GFP-AKT-PH constructs were overexpressed in either HeLa or HEK293T cells. Cells were lysed, and protein extract was diluted in the listed blocking buffer before incubation on PIP strips. No robust ARHGEF26 signal on dotted phosphoinositide species could be detected following immunostaining. Key in bottom right displays location of each phosphoinositide species using a PIP strip incubated with GFP-AKT-PH (also shown in top left) as an example.
