# Supplementary material for: ARHGEF26 enhances Salmonella invasion and inflammation in cells and mice
Source: PLoS Pathog. 2021 Jul 9;17(7):e1009713. doi: 10.1371/journal.ppat.1009713 (PMC8294491; doi:10.1371/journal.ppat.1009713)
Supplement: S3 Fig — DLG1 knockout was confirmed by both immunofluorescence (DLG1 in Gray/Red, Nucleus in Blue, Left) and immunoblotting whole cell lysates (Right). Anti-DLG1 antibody (Santa Cruz Clone 2D11 (sc-9961)) used to detect DLG1. (DOCX) [file ppat.1009713.s004.docx]

**S3 Fig: Confirmation of the DLG1 knockout MDCK cells.** DLG1 knockout was confirmed by both immunofluorescence (DLG1 in Gray/Red, Nucleus in Blue, Left) and immunoblotting whole cell lysates (Right). Anti-DLG1 antibody (Santa Cruz Clone 2D11 (sc-9961)) used to detect DLG1.
