# Supplementary material for: ARHGEF26 enhances Salmonella invasion and inflammation in cells and mice
Source: PLoS Pathog. 2021 Jul 9;17(7):e1009713. doi: 10.1371/journal.ppat.1009713 (PMC8294491; doi:10.1371/journal.ppat.1009713)
Supplement: Attachment — Submitted filename: ARHGEF26_ResponseToReviewsFinal.pdf [file ppat.1009713.s006.pdf]

Response to reviews for "**ARHGEF26 enhances Salmonella invasion and inflammation in cells and mice**."

Editor's and reviewer's comments are reproduced below in normal 10pt font, while our responses are in **bold** 11pt font and *italicized* 11pt font when quoting from the text.

Editor Comments:

Dear Dennis,
Thank you very much for submitting your manuscript "ARHGEF26 enhances Salmonella invasion and inflammation in cells and mice" for consideration at PLOS Pathogens. As with all papers reviewed by the journal, your manuscript was reviewed by members of the editorial board and by several independent reviewers. In light of the reviews (below this email), we would like to invite the resubmission of a significantly-revised version that takes into account the reviewers' comments.
There are quite a few experiments suggested by the three reviewer's to clarify some of your findings. Each point they make is valid. Please pay particular attention to their comments related to the in vivo studies and the recruitment of ARHGEF26 during bacterial-mediated invasion. After reading the manuscript, I have a couple of additional comments.

The authors would like to thank the editors and reviewers for their thoughtful, insightful, and constructive feedback. The substantially revised manuscript that we are submitting has addressed the concerns below and is a significant improvement on our previous submission due to the helpful suggestions provided. Some of the experimental highlights include:

- **Identifying that polarized *ARHGEF26* knockdown and *DLG1* knockout MDCK cells are significantly protected from *S.* Typhimurium invasion in a SopB- SopE2-independent manner**
- **Finding that the differences in *S.* Typhi and *S.* Typhimurium's use of ARHGEF26 during HeLa cell invasion is partially, but not entirely, SopB, SopE, and SopE2 dependent**
- **Determining that the impacts of ARHGEF26 on *S.* Typhimurium fitness in mice require the effectors SopB and SopE2 during the enteric fever model**
- **Discovering that the impacts of SopB and SopE2 on *S.* Typhimurium fitness in mice require ARHGEF26 during the enteric fever model**
- **Demonstrating that the *Arhgef26*$^{-/-}$ inflammation phenotype during the murine colitis model is SopB and SopE2 independent**

Below, each concern is addressed and the resulting changes to the manuscript are provided.
Thank you again,
Dennis Ko, Jeff Bourgeois, *et al.*

(1) Your method of testing "invasion" is unusual. It strictly does not measure invasion (total number of bacteria that were internalised in the monolayer) but rather the percentage of cells that are infected. And it is done at 3 h 15 min post-infection, which is well after the initial invasion event. By this time, Salmonella would have undergone 1-2 rounds of replication inside of cells. As pointed out by one reviewers, your differences in "invasion" are small (<15%). Please confirm whether you get similar results with an assay that quantifies the total number of bacteria that have been internalized e.g. gentamicin protection assay.

This is an interesting point. In our previous manuscripts using this method (1-4) we have used invasion and cell infection synonymously, however, as you point out, we are measuring the host cell-centric process of infection, rather than true bacteria-centric invasion. As such we have made four changes:

1. **Axis titles are changed from "% Invasion (GFP+)" to "% Infected Host Cells (GFP+)"**
2. **Text describing data referring to changes in the % Infected Host Cells now refers to "changes in susceptibility to invasion" rather than to changes in invasion**
3. **The introduction to the flow cytometric method now has the following explanation:** *"Importantly, this assay differs from traditional measures of invasion as it focuses on bacterial uptake as a binary from the host perspective and, as a result, is insensitive to cooperative invasion or early replication events.*"
4. **For our new polarized MDCK phenotype, we examined both the % Infected Host Cells using our flow cytometric method and quantified the actual amount of invasion by confocal microscopy. For this assay, invasion occurred for 30 minutes before the addition of gentamicin and IPTG to inducible GFP and was quantified 2 hours post infection. While there were differences in the size of the impact of ∆SopBE2 on *S.* Typhimurium invasion measured by the two methods, both showed an unequivocal decrease with *ARHGEF26* knockdown, with the microscopic measure of invasion showing a larger effect. This difference is likely explained by the difference in the ability for the techniques to observe cooperative invasion.**

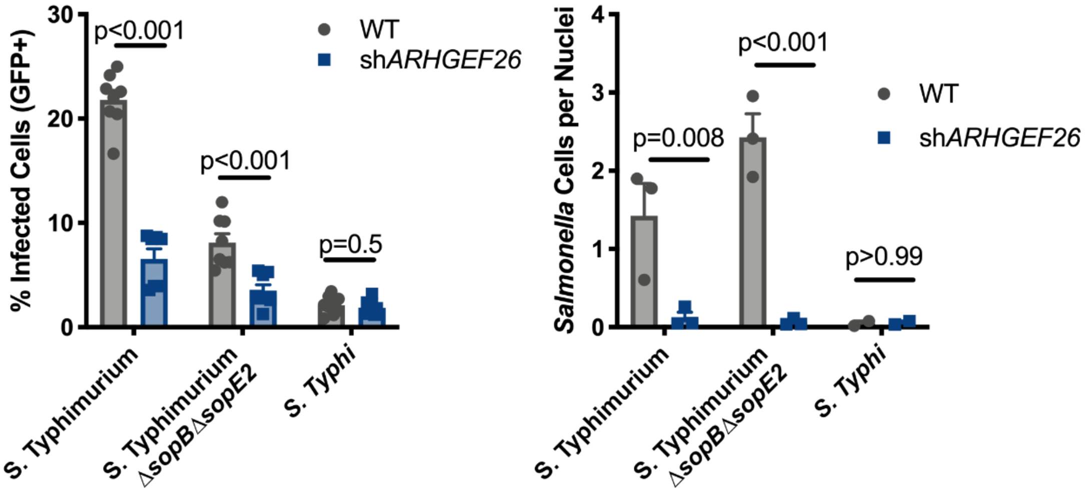

Source: This work (Derived from Figure 6)

Regarding the technical aspects of the modified gentamicin protection assay used in this study, the work presented here and our previous manuscripts (Specifically Alvarez *et al*. (1)) have demonstrated that the assay broadly replicates what has been described with traditional assays, *e.g.*, dramatically reduced rates of host cell infection with ∆*prgH* and SPI-1 effector knockout *Salmonella* (see figures below). However, unlike traditional assays, because this assay uses flow cytometry, moderate effect sizes can be readily

and reproducibly identified by quantifying relatively large numbers of cells (routinely 8000) without any human bias. Thus, we have high confidence in our technique to identify moderate effect sizes, such as those presented in the manuscript.

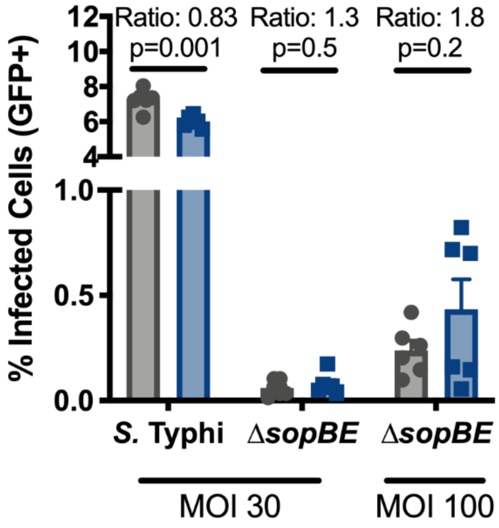

Source: This work (Derived from Figure 3)

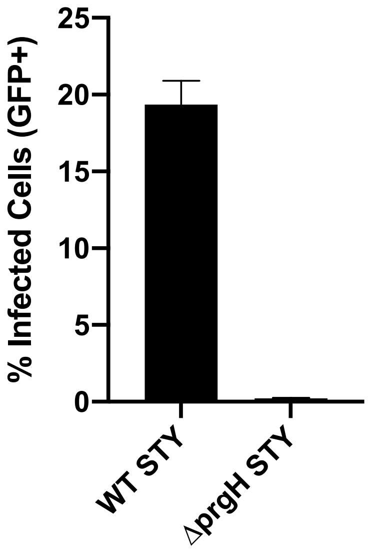

Source: This work (Derived from Figure 5)

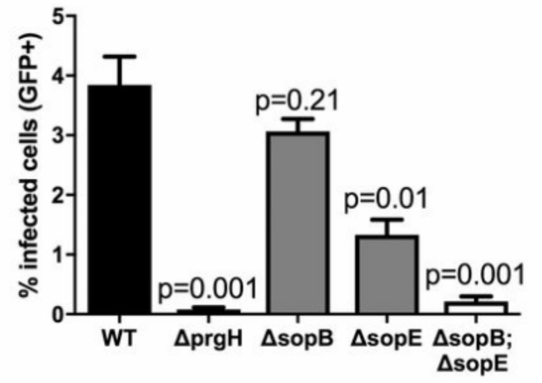

Source: Alvarez…Ko *et al*. 2017

Regarding our time point, we believe that this concern is addressed by altering our language to be host-cell centric, as the flow cytometric method only considers if a cell does or does not contain measurable amounts of GFP-tagged bacteria, *i.e.,* it is agnostic to whether bacterial replication has begun.

(2) The streptomycin-pretreatment mouse model is not a model of gastroenteritis. Rather it should be termed a mouse colitis model. See https://pubmed.ncbi.nlm.nih.gov/21343352/ for clarification. Please correct this throughout, including in figures.

We have corrected the text and figures accordingly.

Reviewer's Responses to Questions:
Part I - Summary
Please use this section to discuss strengths/weaknesses of study, novelty/significance, general execution and scholarship.

Reviewer #1: It has been known for many years that pathogenic Salmonella serovars manipulate host cell GTPases to drive bacterial internalization into non-phagocytic epithelial cells. Previous work has suggested that one of these GTPases is RhoG, activated by the host GEF ARHGEF26 (also known as SGEF) in response to phosphoinositide changes induced by the bacterial effector protein SopB. Here the authors identified ARHGEF26 in a focused genetic screen for natural genetic variants that enhance the infection of lymphoblastoid cells by S. Typhi or S. Typhimurium. Using a variety of in vitro and in vivo (mouse) assays, they demonstrate a small but statistically significant impact of ARHGEF26 on both bacterial internalization and the induction of inflammatory signaling (IL-8 production). In vitro, knockdown of ARHGEF26 appears to selectively affect S. Typhi as opposed to S. Typhimurium, presumably due to differences in the arrays of secreted effector proteins produced by each serovar. In contrast, the most physiologically significant difference is seen in mouse models of S. Typhimurium infection, where knockout of ARHGEF26 inhibits colonization of the ileum (in an enteric fever model) or inflammation, but not colonization in the colon (in a gastroenteritis model).

While the data certainly suggest a potential genetic basis for differential susceptibility to Salmonella infection, no clear unifying mechanism is identified. There are many “plausible hypotheses” but no firm conclusions. The in vitro data indicate that RhoG is not involved in invasion (contradicting an earlier study from the Galan lab), but that the bacterially encoded Rho-GEF SopE is, however no link between ARHGEF26 and SopE or its downstream targets Rac1 and Cdc42 is established. The data also implicate two scaffolding proteins, DLG1 and SCRIB, which are involved in the establishment and maintenance of epithelial junctions, however how they contribute to invasion is never defined, or even explored.

Through this work we have redefined the interactions of ARHGEF26 with *S.* Typhimurium and *S.* Typhi effectors during invasion. Previous work on ARHGEF26 has been limited to a single figure speculating that ARHGEF26 and SopE/SopE2 are interchangeable to

activate RHOG and induce membrane ruffling (5). In this work, we have expanded and substantially revised this model to demonstrate that ARHGEF26 also contributes to SopE-mediated and RHOG-independent invasion of HeLa cells, as well as SopB- SopE2-independent invasion of polarized MDCK cells. Given that this represents a significant difference from past literature, we are specifically very careful to avoid over interpretation of this data in order to encourage future work on this system and avoid speculative mechanisms being recorded as fact. That is to say, the transparency in what we have and have not experimentally demonstrated is intentional. The biology discovered in this work using 3 cellular and 2 mouse models, particularly the large effects observed on invasion into polarized MDCK cells and bacterial burden and inflammation in mice, reveal a central role for ARHGEF26 in *Salmonella* infection. While we agree that it will be useful for the field to further understand the mechanisms by which bacterial effectors, ARHGEF26, and RHO-GTPases connect, many of these questions are outside the scope of this paper and require highly technical cell biology approaches that can be utilized in future work.

Similarly, regarding SCRIB and/or DLG1, our contributions demonstrate SCRIB and DLG1 are required for HeLa cells and polarized MDCK cells, with knockdown or knockout nearly perfectly phenocopying ARHGEF26 knockdown in each situation tested. This is a critical leap forward in understanding ARHGEF26's contributions to invasion, as it ties the observations in *Salmonella* into the current paradigm shifts of ARHGEF26 regulation. Recent work from one of our labs has not only demonstrated that ARHGEF26 requires SCRIB and DLG1 interactions, but that the way in which these three proteins cooperatively regulate different cellular functions varies substantially depending on the specific context being studied (6). In that work, it is established that there are GEF-dependent and a poorly understood GEF-independent roles for the protein complex. Given the significant heterogeneity we observe in our results with complex host by serovar by effector protein interactions, we hypothesize that it is very likely that ARHGEF26, SCRIB, and DLG1 may be performing dramatically different functions across our systems. Therefore, as above, we have defined a new, critical area where gaining greater understanding of these roles represents a very important future direction that is outside of the scope of the work presented here.

Most of the phenotypic effects of ARHGEF26 depletion in vitro are relatively small, suggesting that, whatever its function, it is not a primary driver of invasion or inflammation but may contribute in some secondary way.

The effects observed in vivo are somewhat larger, but confusing and somewhat contradictory. While depletion of ARHGEF26 had no effect at all on S. Typhimurium invasion in cultured cells, it had a significant effect in the ileum during oral infection of ARHGEF26-deficient mice with S. Typhimurium. It is possible that the interacting partners DLG1 and SCRIB have more important roles in the context of a polarized epithelium (i.e. the ileum) than in non-polarized cells in culture, but this issue is never explored.

A key finding of our paper is that the effect of ARHGEF26 on invasion varies substantially depending on the system used. In our previous submission, LCLs demonstrated a 10% proportional decrease in *S.* Typhimurium invasion susceptibility, while HeLa cells demonstrated no difference in *S.* Typhimurium invasion following knockdown. As the reviewer noted, in contrast to these results, the effect in vivo effect on burden was substantial (an approximately 2 log decrease in the ileum in the enteric fever model). This variation in effect size was somewhat puzzling but has now been resolved in following the Reviewer's suggestion to examine the role of ARHGEF26 in

polarized epithelium. In polarized MDCK cells, we observed an 70% proportional decrease in the number of infected cells with ARHGEF26 knockdown, and a 92% reduction in relative invasion as measured by microscopy. While these results are not perfectly representative of the phenomena happening *in vivo* as the effects are SopBE2 independent in MDCKs but dependent in mice, it does set a precedent that ARHGEF26 can have substantial impacts on invasion susceptibility *in vitro*. We thank the reviewer for their suggestion to look at ARHGEF26 function in polarized epithelial cells and believe these experiments have substantially improved the paper.

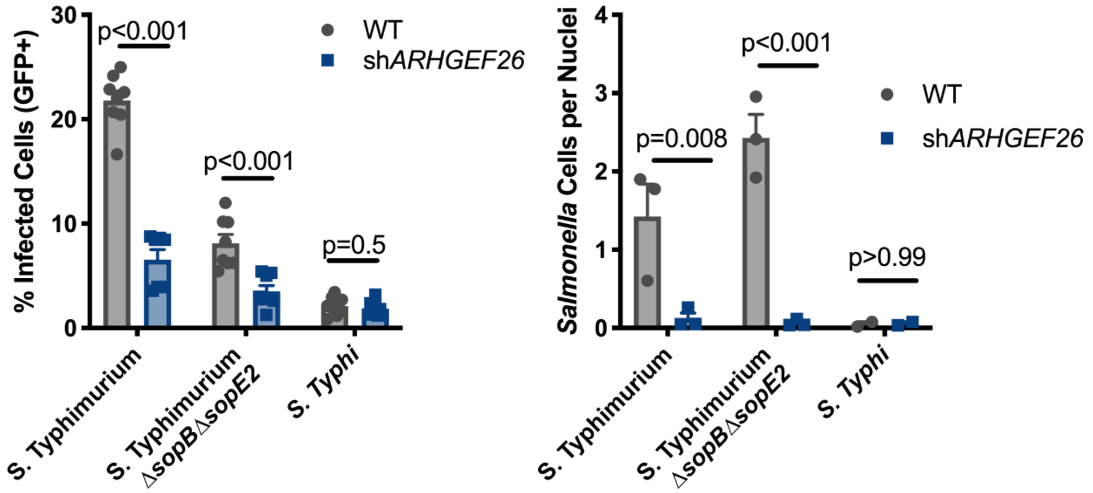

Source: This work (Derived from Figure 6)

In a different model of oral infection (streptomycin pre-treatment) where colonization occurs primarily in the colon, not the ileum, the data indicate no difference in colonization but a significant difference in inflammation (reduced in the absence of ARHGEF26). Although it is possible that regional differences in the intestinal epithelium itself (or regional expression of ARHGEF26) could account for this, the use of two different models of infection makes the result impossible to interpret from a mechanistic perspective.

We apologize for the confusion, as it appears our description of the two mouse models and, in particular, the implications of the equal bacterial burdens at two days post infection in the colitis model, may have caused confusion. The two models should be considered and interpreted separately, as one is a model for enteric fever and the other a model for colitis. We have expanded our description to clarify these points:

"To test whether ARHGEF26 contributes to Salmonella-induced inflammation in vivo, we again examined Arhgef26-/- mice. Importantly, while the enteric fever model is able to measure SPI-1- and invasion-dependent Salmonella fitness in vivo, it does not mimic the natural progression of most S. Typhimurium illness in humans as it causes very little inflammation. In order to mimic S. Typhimurium colitis disease in murine models, microbiota must be reduced with streptomycin pretreatment prior to infection (11) (Figure 9A). Interestingly, in this model, SPI-1 secretion does not impact bacterial burden at early timepoints but instead is required for inflammation onset (10, 11). These differences mean that data from the colitis model cannot be combined with data from the enteric fever model, but that interpreting the colitis model separately can provide important information about pathogenic processes that do not occur in the enteric fever model."

The reduction of inflammation in the *Arhgef26*$^{-/-}$ cecum and colon following infection suggests that the knockout does have an effect on *Salmonella* pathogenesis in these tissues. We propose that *Arhgef26* has tissue- and model-dependent roles during infection, and that not all of these roles are involved in bacterial abundance. In fact, these roles perfectly mimic the effects of SPI-1 ablation in each model. In the enteric fever model where spread and burden in the ileum depends on successful SPI-1-mediated invasion, we see differences in bacterial burden. In contrast, in the colitis model where SPI-1 secretion primarily drives inflammation without impacting burden at early timepoints, we observe differences in pathology. A comparable phenomenon is observed in our *in vitro* data where *ARHGEF26* knockdown reduces IL-8 production following *S.* Typhimurium infection without impacting HeLa cell invasion. These pleiotropic effects are consistent with SPI-1 and ARHGEF26 having separate roles in invasion or inflammation depending on the *in vivo* model used.

Reviewer #2: Through the GWAS screen, Bourgeois et al. identified ARHGEF26 (also known as SGEF) as a host factor that up-regulates Salmonella invasion. In vitro invasion assays using HeLa cells, they found that ARHGEF26 contributes to SopB- and SopE-mediated S. Typhi (but not S.Typhimurium) invasion. They examined the molecular mechanism how ARHGEF26 activity is regulated, focusing on the ARHGEF interactors, DLG1 and SCRIB, as well as by the ARHGEF26 domain analysis. They further showed the possible role of ARHGEF26 in Salmonella-induced inflammation by monitoring IL-18 secretion. Finally, they demonstrated the contribution of ARHGEF26 to S. Typhimurium fitness using the mouse model.

Overall, the manuscript is well-written and gives new insights into a role of ARHGEF26 in SopE-mediated Salmonella invasion and inflammation, although there are some inconsistencies between results depending on the experimental systems (ex. HeLa cells vs LCLs) as the authors discussed. For the disagreement with a previous report, I think that some results need to be more clarified by conducting additional experiments.

Thank you for your positive feedback. We would like to state that while our report disagrees with the canonical roles of ARHGEF26 and RHOG during *Salmonella* uptake, we are not the first to dispute the involvement RHOG in invasion (7, 8). Though, of course, we are happy to improve the rigor of this report through the additional experiments suggested by the reviewers.

Reviewer #3: In this manuscript the author have investigated the role of ARHGEF26 in response to Salmonella Typhi and Salmonella Typhimurium infection. They demonstrate that the effector proteins SopE and SopB of S. Typhi are required for efficient ARHGEF26-dependent invasion in HeLa cells, contrary to S. Typhimurium effectors SopE2 and SopB which appeared not to require ARHGEF26 to invade HeLa cells. They further demonstrate that activation of ARHGEF26 can induce inflammatory responses, and that ARHGEF26 plays a critical role in S. Typhimurium pathogenesis in both the enteric fever murine model and the murine gastroenteritis infection model.

Strengths:
Natural genetic variations in ARHGEF26 were identified in a cellular GWAS screen that contribute to susceptibility to Salmonella Typhi and Typhimurium invasion.
Demonstrated new roles of ARHGEF26 during Salmonella invasion.
Strong in vitro and in vivo data with a great deal of experimental repeats.

Weaknesses:
Some controls that will strengthen the conclusions are missing.
Additional time points in the in vivo experiments should be included.

Thank you for your informative feedback and for your complimentary assessment of the manuscript.

Part II – Major Issues: Key Experiments Required for Acceptance
Please use this section to detail the key new experiments or modifications of existing experiments that should be absolutely required to validate study conclusions.

Generally, there should be no more than 3 such required experiments or major modifications for a "Major Revision" recommendation. If more than 3 experiments are necessary to validate the study conclusions, then you are encouraged to recommend "Reject".

Reviewer #1: 1. Fig. 2 shows a very small (<15%) reduction in invasion of LCLs by both S. Typhi and S. Typhimurium after knockdown of ARHGEF26. While Fig. 3 shows a slightly higher inhibition of invasion in Hela cells by S. Typhi (~30%), there is no effect on S. Typhimurium. How do the authors explain this discrepancy? Do both cell types express ARHGEF26 at similar levels? What about DLG1 or SCRIB? Are there differences in expression?

This was an interesting hypothesis that we had not previously considered. We have observed that ARHGEF26 amplifies at a lower CT in RNA from HeLa cells. In fact, measuring ARHGEF26 expression in LCLs is often challenging as it is often present in levels at or below the limit of detection of our qPCR assay. Thus, this could explain the differences between the magnitude of effect between the two cell lines. We have added the following sentence in the section where we introduce the HeLa cell data.

"ARHGEF26 RNAi knockdown (~75% reduction, Figure S1F) significantly reduced the proportion of cells susceptible to S. Typhi invasion (Figure 3A). Notably this phenotype was larger than we observed with LCL knockdown. While a number of factors could contribute to this difference, we observed that LCLs expressed ARHGEF26 close to the technical limit of our TaqMan assay, while HeLa cells expressed easily detectable levels of ARHGEF26. We therefore hypothesized that increased ARHGEF26 expression in HeLa cells may promote a larger role for the protein in invasion."

2. Even in Hela cells, knockdown of ARHGEF26, DLG1 or SCRIB yielded only small differences in invasion (Fig. 4). Given the important roles of DLG1 and SCRIB in epithelial junction formation, it is surprising that the authors did not examine invasion in a polarized epithelial cell model (e.g. Caco-2 or HT-29 cells).

As mentioned above, we are very grateful for this suggestion. We find that ARHGEF26 and DLG1 are all critical for WT *Salmonella* Typhimurium and ∆SopBE2 *S.* Typhimurium uptake into polarized MDCK cells. This data is now included as Figure 6.

3. Dissection of ARHGEF26 suggests that interaction with DLG1 and/or SCRIB is important to its function. Do these interactions stimulate the GEF activity of ARHGEF26?

Previous work (9) has demonstrated that Dlg1 knockdown affects the ability for ARHGEF26 to activate RHOG. The figure from that paper is included below and we have added the following sentence to our description of the DLG1/SCRIB data:

"Previous work has demonstrated that the Scribble complex members DLG1 and/or SCRIB play key roles facilitating ARHGEF26 activity and RHOG activation during human papillomavirus

infection (43), as well as in regulating epithelial junction formation, contractability, and lumen formation in 3D cysts (44).”

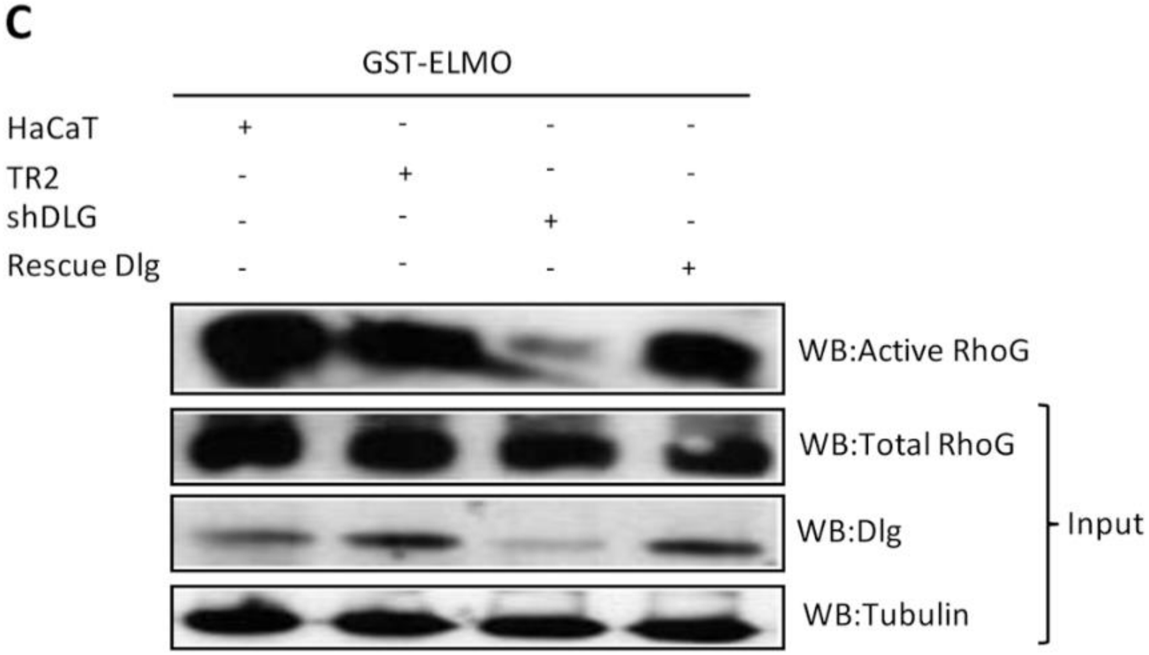

Source: Subbaiah *et al*. 2012. (9)
Source Figure Legend: “Panel C. Extracts from HaCaT cells stably ablated for hDlg expression (shDlg) either with or without Dlg rescue expression were used in a GST-ELMO pulldown assay to determine RhoG activity. Extracts from untreated HaCaT cells or HaCaT cells stably expressing control shRNA(TR2) were used as control.”

However, this has not been demonstrated in the context of *Salmonella.* As our data do not support a role for RHOG in contributing to *Salmonella* invasion, we have opted not to pursue examining how DLG1 and SCRIB contribute to Salmonella-induced RHO-GTPase activity until we have identified the causal GTPase connecting ARHGEF26 and *Salmonella* uptake, which is beyond the scope of this manuscript.

4. As mentioned above, the mouse models yield confusing and contradictory data. One possible reason for this is that the ARHGEF26 knockout is not tissue-specific, and the data could reflect altered responses in the epithelium, stromal cells or infiltrating lymphocytes. It could also reflect differential expression of ARHGEF26 or splice variants in the ileum vs. cecum or colon. Neither of these issues is addressed, and the results are therefore impossible to interpret.

As we responded above, we thank you for raising these concerns about our mouse model descriptions, and we have improved the clarity of the models and their interpretation. The two models should be considered separately, as models of enteric fever and colitis, and each provides novel insight on the critical roles ARHGEF26 has in controlling burden and inflammation during infection.

In the enteric fever model, *Salmonella* invasion plays a critical role in promoting *Salmonella* burden in the ileum (10). In this model, ARHGEF26 deletion causes a ~2-log reduction in *Salmonella* burden in the ileum (Figure 8B). The magnitude of this effect is

equal to the effect of sopB/sopE2 double mutant and is entirely dependent on the presence of these two effectors (Figure 8C). Further, in the absence of ARHGEF26, sopB/sopE2 double mutant has no effect on bacterial burden in the ileum. This result reveals a central role for ARHGEF26 in the function of sopB and sopE2 in the enteric fever mouse model.

In contrast, *Salmonella* SPI-1 mediated invasion does not affect burden in the colitis model at two days post infection. This is demonstrated extensively in other work with bacterial invasion mutants (11, 12) as well as in our own hands, as not only do *Arhgef26*$^{-/-}$ and wild-type mice have equal bacterial burdens in the ileum, cecum, and colon, but so does a SPI-1 mutant (Δ*prgH*).

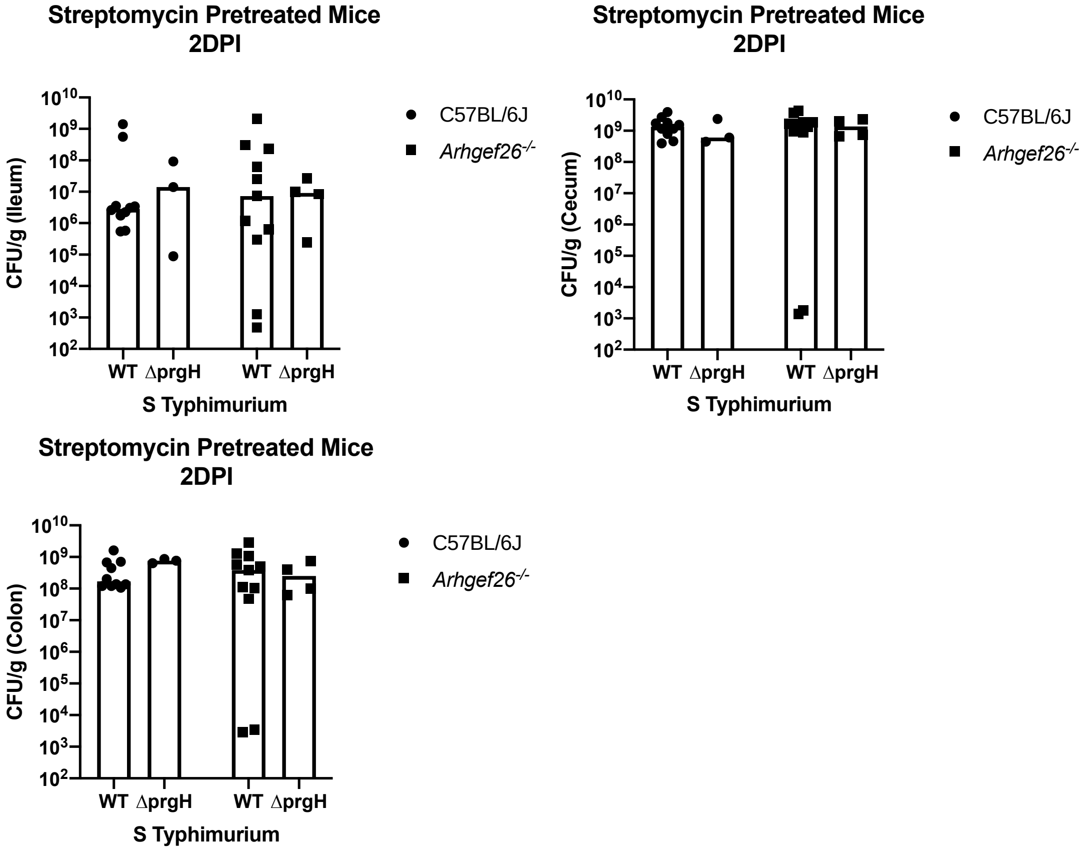

Source: Unpublished Data. Data from the colitis mouse model with streptomycin pretreatment, tissues collected two days post infection. C57BL/6J vs *Arhgef26*$^{-/-}$ wild-type *S.* Typhimurium data are included in Figure 9B.

Thus, SPI-1 mediated invasion is not contributing meaningfully to *Salmonella* burden in any of these tissues in the colitis model. This therefore argues against a hypothesis in which there would be invasion-mediated differences in bacterial burden if there were different levels of expression or different splice variants in these distal tissues. Further, as mentioned above, the differences in inflammation suggest that *Arhgef26* knockout <u>is</u> having an effect in the cecum and colon, as there are significant changes in inflammation.

Interestingly, the hypothesis that variation in ARHGEF26 expression or splicing across tissues may be important becomes better supported in our new data four days post infection in the colitis model. At this timepoint, we do observe a difference in ileal burdens, but do not see differences in burden in the colon or cecum. We have added discussion in light of this in the text and have included it below:

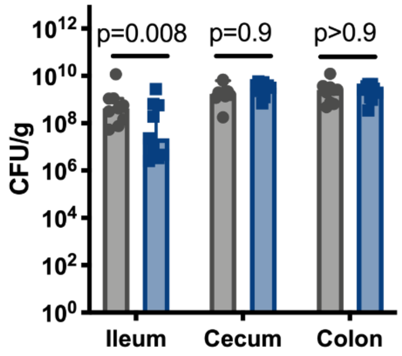

Source: This work (Derived from Figure 9)

"Given our finding that Arhgef26-/- mice have substantially reduced inflammation, we were surprised to find that bacterial burden was not affected in the large intestine at late time points during the colitis model (Figure 9F). We propose four hypotheses for why we observe this result… The fourth hypothesis is that differences in ARHGEF26 expression across the different gastrointestinal compartments could lead to the observation that S. Typhimurium burden is reduced in the ileum, but not the cecum or colon, at four days post infection in the colitis model."

Reviewer #2: (1) The in vitro invasion assay using HeLa cells demonstrated that ARHGEF26 contributes to both SopB- and SopE-mediated S. Typhi invasion (Figure 3), while the previous work (Ref 7) showed that ARHGEF26 is involved in SopB-, but not SopE-mediated (S. Typhimurium) invasion. As the genetic background of Salmonella strains (as well as cell-lines) are different between the two works, it is important to ensure that ARHGEF26 works solely on the SopB/SopE axis in the strains used in this study. The double knockout (ΔsopB ΔsopE) S. Typhi strain (which should give no difference between NT and siARHGEF26) needs to be included in Figure 3D as well as in Figure 4C). This strain may give the passive invasion as observed with ΔprgH (Figure 7C), but should be a required control.

Thank you for your insightful suggestion. In addition to the experiments you suggested, we tested the ΔsopBsopE or ΔsopBsopE2 mutants in a variety of our other experimental systems to examine which conditions do and do not appear to fall along the SopB/SopE/SopE2 axis.

While it is difficult to measure *Salmonella* uptake in *ΔsopBΔsopE S.* Typhi by HeLa cells due to their inefficient invasion, we infected HeLa cells at a high MOI and measured ~0.5% infection, which did not vary between NT and siARHGEF26 conditions, suggesting that in HeLa cells the ARHGEF26 infection decrease is occurring by SopB/SopE-mediated mechanisms.

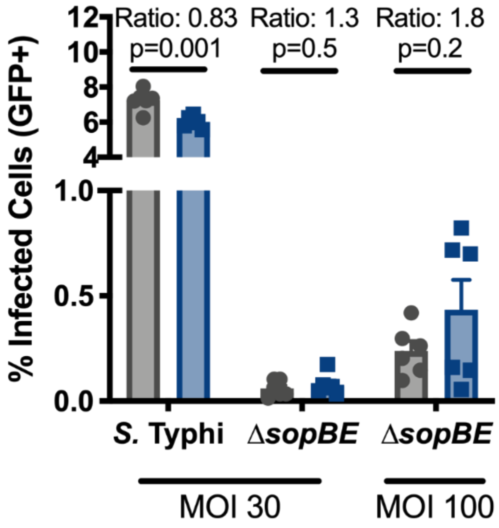

Source: This work (Derived from Figure 3)

In addition to this experiment, we performed an experiment suggested by reviewer three to test whether moving the *sopB* and *sopE2* genes from *S.* Typhimurium into *S.* Typhi could cause *S.* Typhi to lose its sensitivity to host ARHGEF26 knockdown. Curiously, we found that expressing *S.* Typhimurium SopB rendered *S.* Typhi sensitive to ARHGEF26, while *S.* Typhi expressing SopE2 actually invaded siARHGEF26 transfected cells better than NT transfected cells. Given that knocking out *sopE2* does not render *S.* Typhimurium resistant to ARHGEF26 mediated invasion, we conclude that the effects of ARHGEF26 on HeLa cell invasion is only partially dependent on the SopB/SopE/SopE2 effectors and that other effectors also determine whether *Salmonella* strains will be sensitive to HeLa cell ARHGEF26 knockdown.

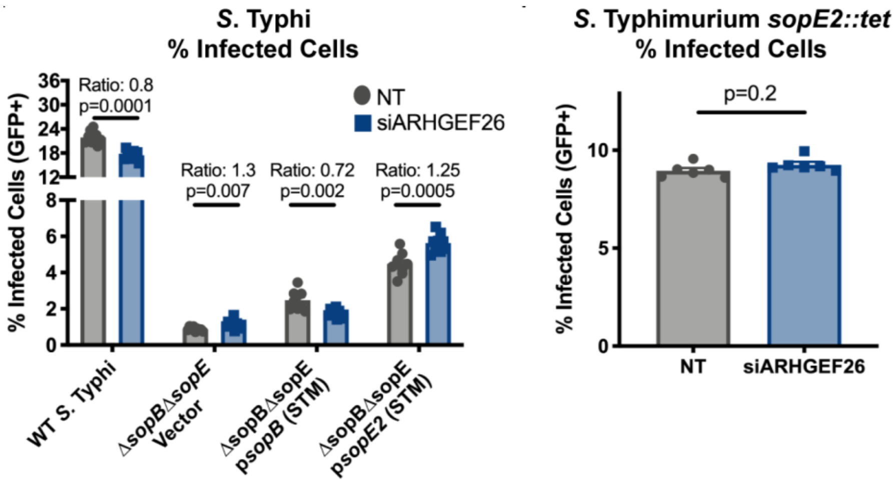

Source: This work (Derived from Figure 3)

In contrast, in polarized epithelia, we find that the ARHGEF26 and DLG1 *S.* Typhimurium phenotypes are completely independent of SopB and SopE2, as the invasion deficits are present regardless of the two effectors.

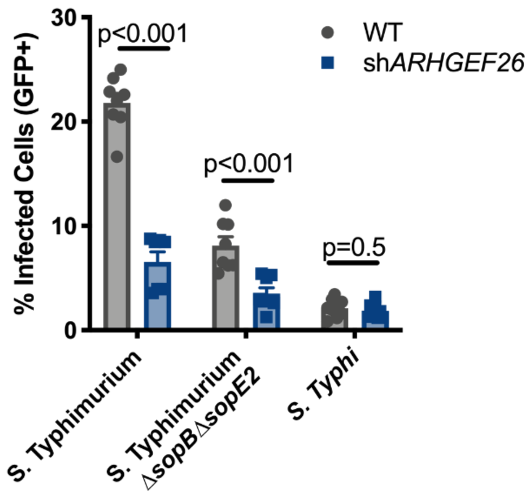

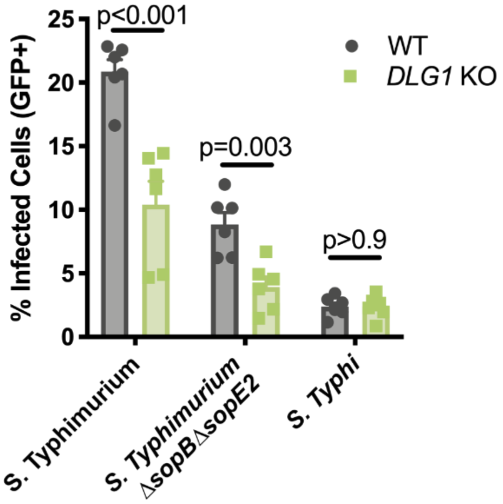

Source: This work (Derived from Figure 6)

Further, in our murine enteric fever model, the effects of ARHGEF26 on *S.* Typhimurium fitness depended on SopB and SopE2. Shockingly, we also found that the effects of SopB and SopE2, in turn, require ARHGEF26.

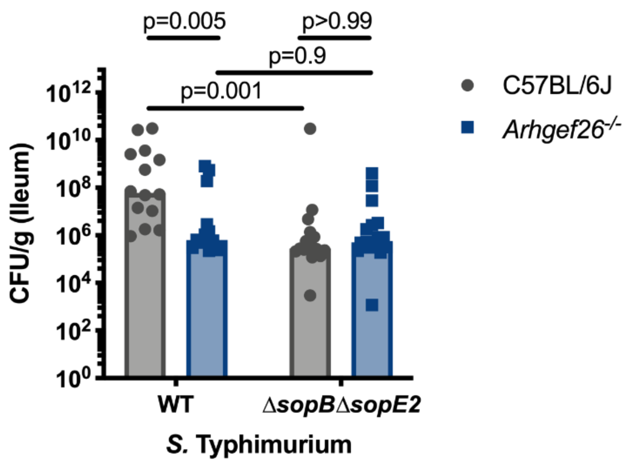

Source: This work (Derived from Figure 7)

Finally, we show that in our colitis murine model, our inflammation phenotype is SopB and SopE2 independent.

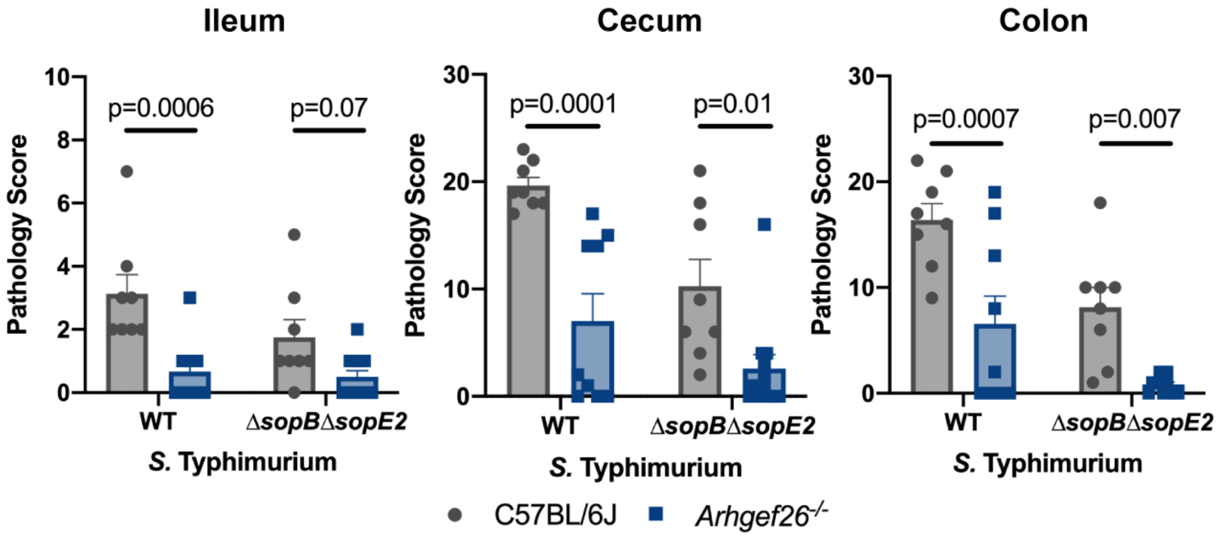

Source: This work (Derived from Figure 9)

Together, these data demonstrate a fascinating and complex relationship between ARHGEF26/DLG1/SCRIB and SopB/SopE/SopE2. In some contexts, particularly in HeLa cells and the enteric fever murine model, SopB/E/E2 appear to be critical for ARHGEF26 manipulation and infection. In other systems, particularly in polarized MDCKs (which have recently been described to partially invade by SopB/SopE/SopE2 independent mechanisms (13)) and regulating inflammation, ARHGEF26's contributions appear to be SopB/SopE2 independent. We have added these data and discussion of these points throughout the text.

(2) (Ref 48), detail of the molecular interaction between ARHGEF26 and DLG1/SCRIB has been analyzed. DLG1- and SCRIB-binding regions seem to locate just upstream of the DH domain of ARHGEF26. I wonder if the 415-871 construct in Figure 5A possesses the ability to interact with DLG1- and SCRIB. As the authors propose that SCRIB-DLG1-ARHGEF26 complex guides the GEF to the plasma membrane (lines 699-700), the interaction should be disrupted experimentally by introducing mutations in the binding regions to see if the interaction is essential for enhancing invasion.

We have wanted to perform this experiment for some time, however, the 415-871 construct still retains high ruffling and invasion activity, which we feel is a non-physiological artifact of overexpression. We show that the proposed mutant (415-871), despite not being able to interact with DLG1 and SCRIB (6), still causes spontaneous membrane ruffling (which we hypothesize is due to its intact catalytic domain and accidental interactions with RHOG and other GEFs that occur in the overexpression system regardless of specific localization) and uptake of non-invasive bacteria.

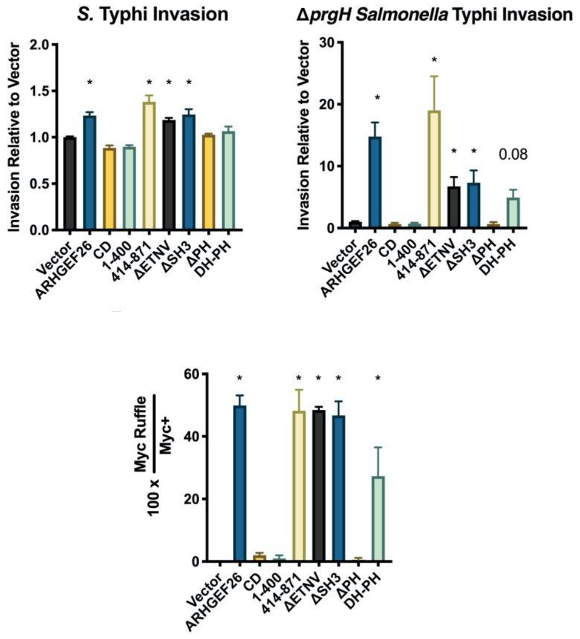

Source: This Work (Figure 5)

Therefore, because bacterial uptake and spontaneous membrane ruffling cannot be decoupled in this system (Figure 5), we cannot determine the impact of the ARHGEF26 N-terminus on invasion without direct manipulation of the host genome, which would allow assessing the terminus' function without driving spontaneous membrane ruffling through overexpression. This will be an interesting future direction but is outside the scope of this study.

(3) For clarifying the role of ARHGEH26 in Salmonella invasion, it is crucial to demonstrate the recruitment of the protein to the site of invasion. Infection experiments can be conducted using HeLa cells expressing Myc-tagged ARHGEF26 and its derivatives, not only showing transfection-induced ARHGEF26-positive membrane ruffles (Figure 5DE).

Once again, we have tried multiple approaches to address this question both before the original submission and during the revision process in light of your review. Regardless of the approach used, we have found that the overexpression system makes it impossible to distinguish between enrichment of ARHGEF26 at the membrane ruffle and the high amounts of ARHGEF26 present in the cytosol (which fills the *Salmonella* induced ruffle). We have reached this conclusion by overexpressing mCherry and comparing localization to a catalytically dead ARHGEF26-mCherry construct. The catalytically dead construct was chosen because the wild-type construct causes such widespread spontaneous ruffling that *Salmonella* induced ruffles cannot be distinguished.

As you can see below, with confocal microscopy we see near equal presence of the mCherry florescent protein at the membrane ruffle. Thus, it would be misleading to draw any conclusions about localization from our overexpression system.

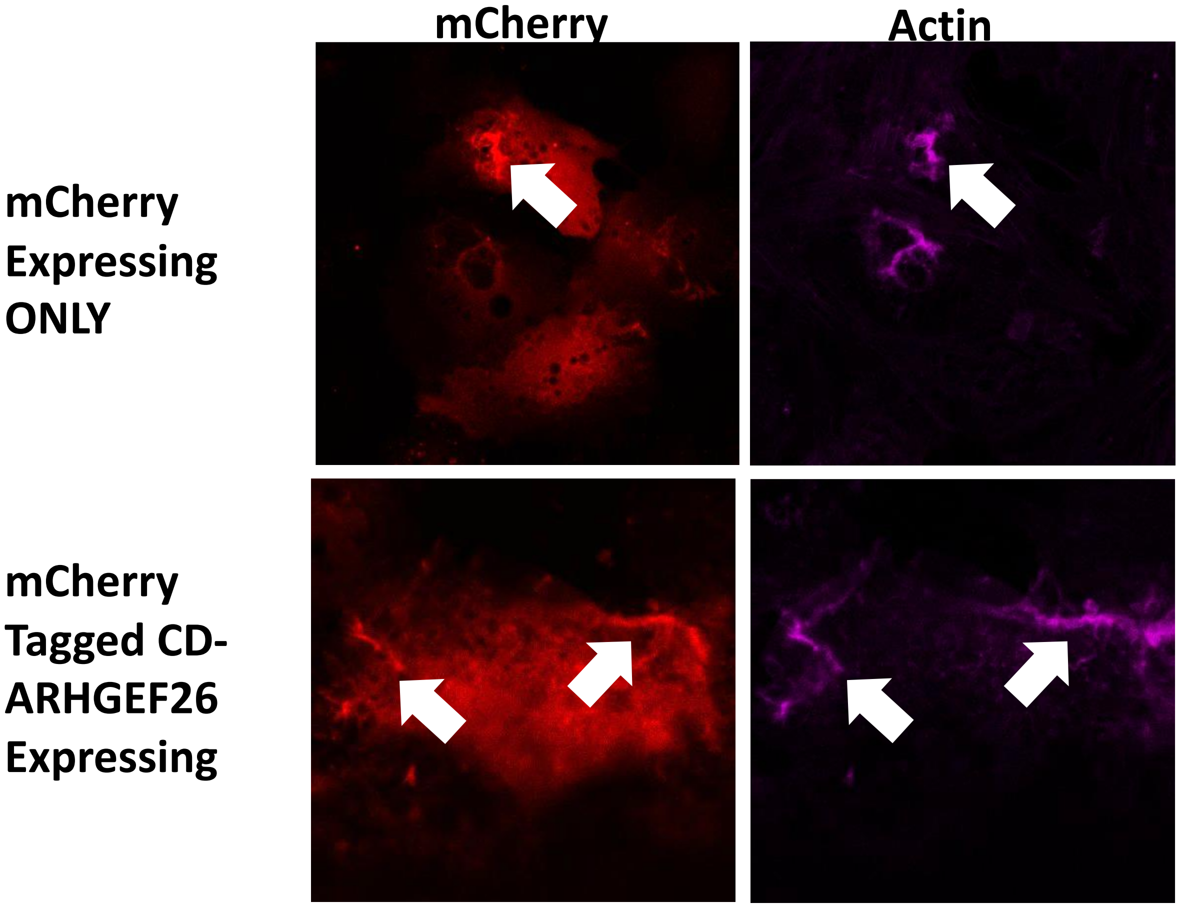

Source: Unpublished Data. Cells expressing mCherry-C1 or an mCherry tagged CD-ARHGEF26 construct were infected with *S.* Typhi for 30 minutes, immediately fixed with 4% PFA and 1% glutaraldehyde before mounting. Actin was stained with Alexa Fluor™ 647 Phalloidin (Thermo). Micrographs were taken with the 63x objective on a Zeiss LSM 510 confocal microscope. Z-stacks were compressed into Z-projections by sum slices using Fiji (14).

Given this finding, we conclude that advanced cell biology and genetic techniques to localize endogenous levels of a low abundance protein will be required to understand exactly how ARHGEF26 contributes to invasion, and which domains are involved in that process. While an important question, we believe the complexity of this question leads it to be outside the scope of this paper.

Despite the technical limitations, your suggestion is well taken, and we have removed all mention of DLG1 and SCRIB directing ARHGEF26 to the ruffle. Thus, our discussion of ARHGEF26, DLG1, and SCRIB throughout the paper more accurately reflect our findings.

Reviewer #3: 1. The authors show that ARHGEF26 is not required for S. Tm SopB-dependent invasion, and speculate that a modest difference in sequence between SopB of S. Tm and S. Typhi might account for the differential ARHGEF26 requirement. Have the authors considered complementing a S. Tm DsopE2/sopB double mutant with S. Typhi SopB and/or SopE to investigate whether the ARHGEF26 requirement is dependent on SopB and/or SopE of S. Typhi and not caused by other differences in the invasion mechanisms of S. Typhi and S. Tm? The reverse can also be achieved; complement S. Typhi mutants with S. Tm SopB and/or SopE2 to clearly pinpoint whether these effector proteins contribute (or not) to ARHGEF26-dependent invasion in HeLa cells.

This was a fascinating suggestion that we took great joy in executing. We opted to perform the second described experiment due to the availability of plasmids and strains. Curiously, we found that complementing *S.* Typhi with *S.* Typhimurium SopB did not impact the ability for *ARHGEF26* knockdown to impact *S.* Typhi invasion. In contrast, complementing SopE2 did overcome *ARHGEF26* knockdown and actually resulted in increased invasion compared to non-targeting. This was slightly unexpected, as we showed in the original submission of this manuscript that knocking out SopE2 in *S.* Typhimurium did not restore ARHGEF26 sensitivity. Together, these data demonstrate that that while the SopE vs SopE2 effector regiments in this study help to explain differences in strain dependence on *ARHGEF26* in HeLa cells, they are not the only contributors to the phenotype.

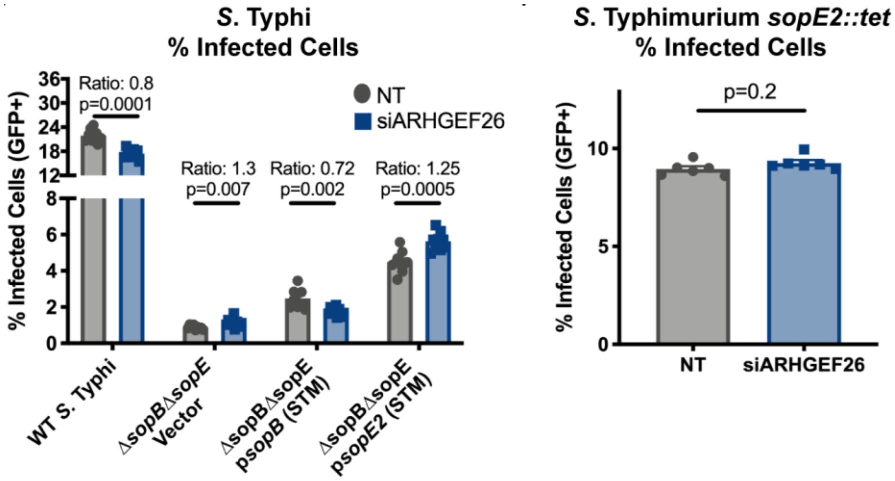

Source: This work (Derived from Figure 3)

2. The in vivo role of ARHGEF26 was determined by infecting Arhgef26-/- mice using two different mouse models, the enteric fever murine model and the murine gastroenteritis infection model. Arhgef26-/-mice have reduced bacterial numbers in ileum and spleen (Fig 7), and this is dependent on a functional T3SS-1, since a DprgH mutant no longer showed reduced bacterial numbers in Arhgef26-/- mice compared to wild type mice. A DsopB mutant, however, still had significantly lower numbers in the ileum, suggesting S. Tm SopB does not contribute to ARHGEF26-dependent bacterial colonization. It is unclear why the authors state that this is dependent on SopB (lines 601). Is this conclusion solely based on the reduced ratio (0.04) compared to mice infected with wild type Salmonella (0.004)? What is the bacterial load of the DsopB mutant in the spleen of wild type and Arhgef26-/- mice? And does SopE play a significant role in the murine enteric fever model?

Thank you for pointing out that this is unclear in the text. Your interpretation is correct that we were referring to the change in ratio. However, in light of our new data showing that the phenotype is dependent on SopB and SopE2, we agree that referring to the ARHGEF26 phenotype as being partially SopB-dependent based on this minor shift is distracting. We have now revised the text to reflect this.

"Supporting that a functional SPI-1 secretion system is required for ARHGEF26 to affect S. Typhimurium fitness, the difference in burden between wild-type and Arhgef26-/- mice was significantly reduced when mice were infected with ΔprgH bacteria (Figure 7C). In contrast, differences between wild-type and Arhgef26-/- mice were slightly reduced but broadly maintained when infected with a ΔsopB S. Typhimurium strain, once again demonstrating that

the previous model that ARHGEF26 contributes specifically to SopB-mediated fitness is incomplete.”

Regarding the spleen, the phenotype in the spleen is very small given the noise we observe and is therefore very difficult to make mechanistic conclusions about, which is why we did not include the data displayed below in the manuscript. With the moderate number of mice used in these experiments we did not see statistically significant differences or ratios resembling what we see in ileum where the phenotype is substantially larger. Further, the very well-defined role for SPI-1 in the ileum and not systemic sites makes the ileum the more relevant site to examine for sopB dependence.

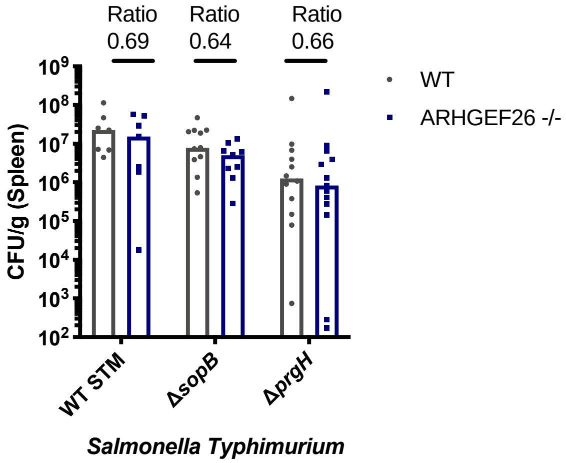

Source: Unpublished data, paired with Figure 7C

Furthermore, in light of your suggestion and suggestions for Reviewer 2, we have performed experiments using the ∆sopB∆sopE2 *S.* Typhimurium mutant. Excitingly, we found that the entire ARHGEF26 phenotype depends on ∆sopB∆sopE2. Perhaps even more unexpected, we found that, in turn, the entire ∆sopB∆sopE2 phenotype depends on ARHGEF26. This suggests that despite the GEF activity of SopE2, it requires ARHGEF26 involvement in order to induce *S.* Typhimurium fitness in the ileum.

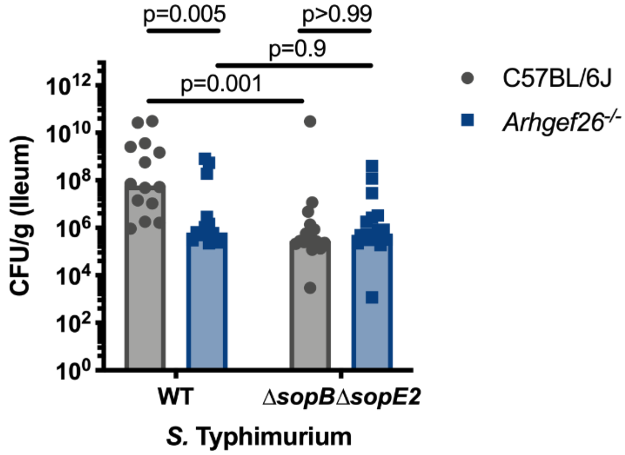

Source: This work (Derived from Figure 7)

3. In the gastroenteritis model is there a reduction in cytokine (eg. KC) production that would correlate with the findings in HeLa cells?
It has been demonstrated that Salmonella-induced inflammation is beneficial for Salmonella to grow to high numbers. Does the reduction in total pathology scores result in reduced bacterial colonization in the Arhgef26-/- mice at later days post infection (days 3 and 4)? And do the effector proteins SopB and/or SopE contribute this ARHGEF26-dependent inflammation?

Due to the COVID-19 pandemic, we have been limited in the amount of mouse breeding that was able to occur over the last 15 months. To try to minimize the numbers of mice we needed for this revision process, we attempted to collect fecal biomarkers of inflammation that would allow us to measure inflammation while also measuring bacterial burden and/or pathology. Therefore, in order to address this suggestion, we examined secreted lipocalin-2 (15). Unfortunately, we did not observe statistically differences between wild-type and ARHGEF26 mice at two days post infection due to very high variation in the assay. We predict that if we had measured luminal cytokines in sufficiently large numbers, we would have been able to generate corroborating data for the pathology differences which were collected in a blinded fashion and are unequivocal.

Colitis Model: Two Days Post Infection

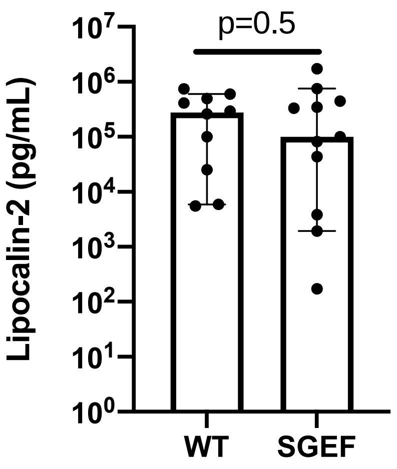

Source: Unpublished data. Lipocalin-2 measured from feces collected over a 12 hour period ending at 2 days post infection. Lipocolin-2 measured by ELISA (R&D Systems)

Regarding the later timepoint for CFUs, surprisingly, we did not observe differences in bacterial burden at four days post infection in the cecum or colon despite the differences in inflammation.

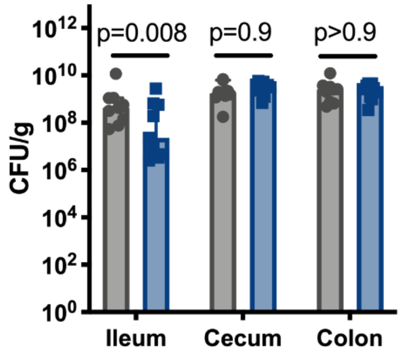

Source: This work (Derived from Figure 9)

In the text we provide a number of explanations for why we observed this result, and have reproduced that section here:

"Given our finding that Arhgef26-/- mice have substantially reduced inflammation, we were surprised to find that bacterial burden was not affected in the large intestine at late time points during the colitis model (Figure 9F). We propose four hypotheses for why we observe this result. The first is that compared to previous work on inflammation-promoted bacterial burden (65), we used a higher inoculum of S. Typhimurium and that at this dose S. Typhimurium may be able to overcome modest microbiota recolonization. The second is that compared to the avirulent S. Typhimurium strain used in that study, Arhgef26-/- may be able to produce enough inflammation to prevent microbiota recolonization. The third hypothesis is that while we observed substantially reduced inflammation at two days post infection by pathology scoring, Arhgef26-/- mice may have altered dynamics of inflammation, rather than outright ablation of inflammation. Thus, at four days post infection, Arhgef26 knockout mice may have elevated inflammation that can clear out recolonizing microbes. The fourth hypothesis is that differences in ARHGEF26 expression across the different gastrointestinal compartments could lead to the observation that S. Typhimurium burden is reduced in the ileum, but not the cecum or colon, at four days post infection in the colitis model."

Finally, regarding your question regarding ∆*sopB*∆*sopE2* influencing the colitis model phenotype, we find that ARHGEF26 actually reduces pathology in all three tissues (ileum, cecum, colon) more than we observed ∆*sopB*∆*sopE2* reducing pathology, and that these reductions appear to be at least largely independent (full independence cannot be assessed due to reaching the limit of detection of the assay).

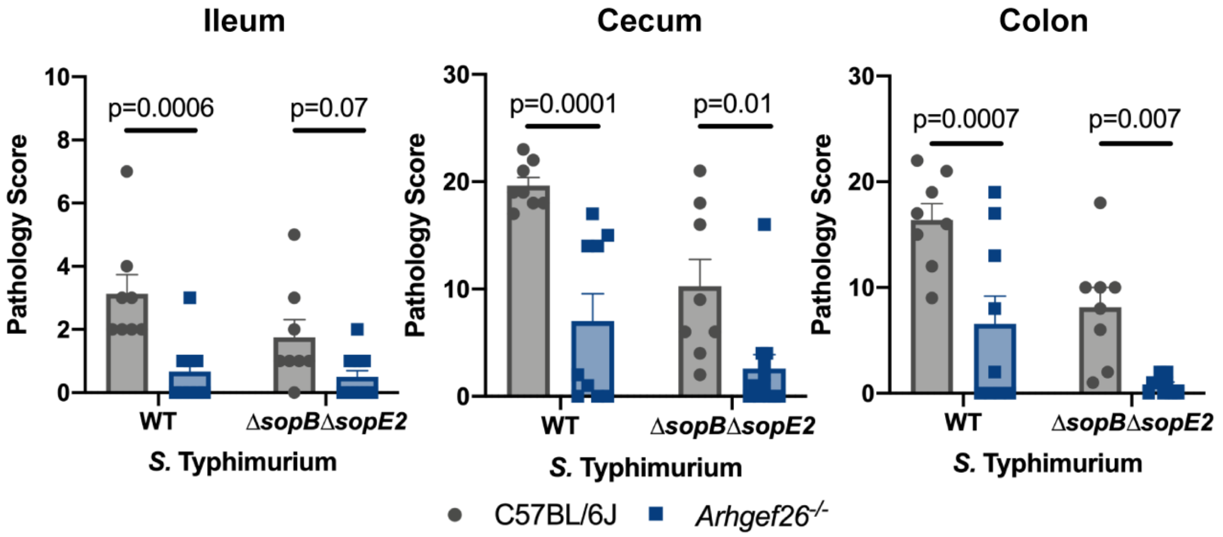

Source: This work (Derived from Figure 9)

Part III – Minor Issues: Editorial and Data Presentation Modifications
Please use this section for editorial suggestions as well as relatively minor modifications of existing data that would enhance clarity.

Reviewer #1: (No Response)

Reviewer #2: (1) I wonder why the ΔsopB S. Typhi strain shows the higher invasion late than the wild-type strain in HeLa infection experiments (Figure 3D, Figure 4CD). Are there any reasonable explanations?

We have observed that the ΔsopB mutant typically invades equal to slightly higher than the wild-type strain, though occasionally we do see less invasion. Figures 3D (Now Figure 3B) and the experiments for Dlg1 knockdown in 4C were from the same experiments where ΔsopB did, by chance, have some of the most consistent increased invasion we've seen. As a result, it did have a significant p-value by a t-test.

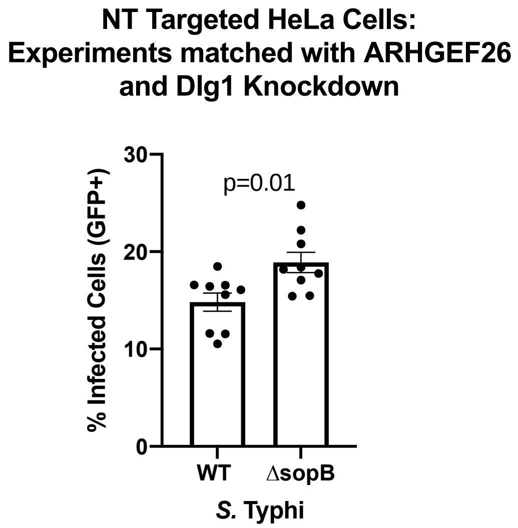

Source: This work (Derived from Figure 4)

By contrast, the experiments matched with siSCRIB knockdown did not meet significance, though there was slightly smaller N for these experiments (6 replicates per group vs 9 in the experiment above)

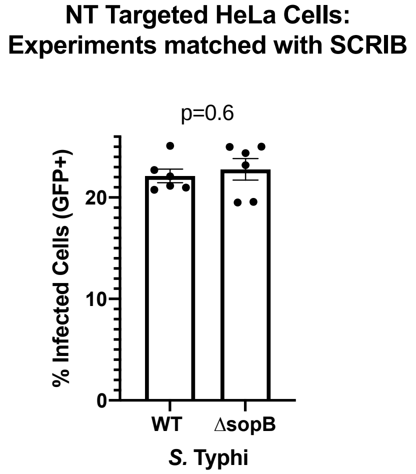

Source: This work (Derived from Figure 4)

Because the phenotype is, typically, not consistent, we do not comment on the phenotype in the study. From a biological perspective, knocking out sopB may allow for a greater amount of secretion of other effectors, such as sopE2, that may be better at stimulating invasion.

(2) The authors did not get experimental data supporting that RhoG has any roles in Salmonella invasion and in "infection-induced" IL-8 production. I wonder if the authors think that the genetic diversity of SopE in the Salmonella strains can cause the discrepancy between studies (lines 720-722).

This idea of SopE/E2 diversity contributing to phenotypes observed in this study was explored through a major experiment suggested by reviewer three above. We "switched" the *S.* Typhimurium *sopE2* gene into *S.* Typhi and found that it did render *S.* Typhi

insensitive to host ARHGEF26 knockdown-mediated invasion resistance. However, knocking out SopE2 in *S.* Typhimurium did not restore ARHGEF26 sensitivity, suggesting that while SopE/E2 diversity might help to explain differences in strain dependence on *ARHGEF26* in HeLa cells, it is not the only contributor.

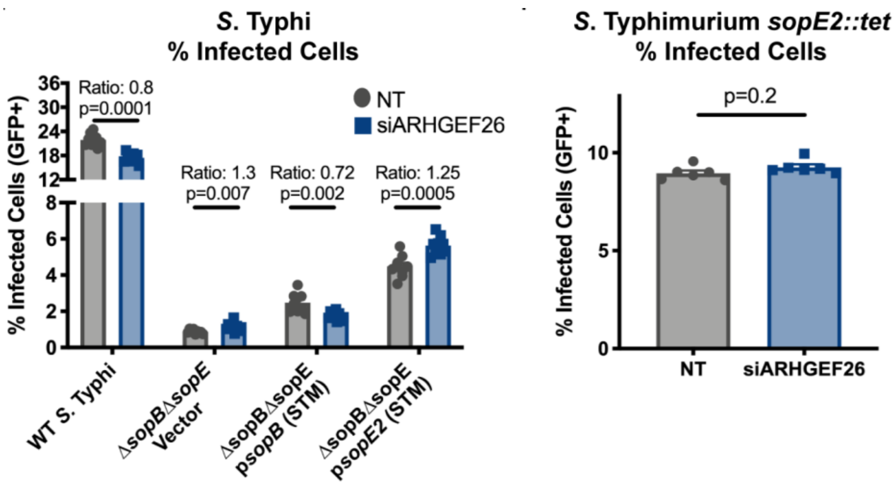

Source: This work (Derived from Figure 3)

In terms of RHOG, SopE/E2 diversity is unlikely to explain differences between our study and the Patel and Galán 2006 study, as in their work they used both wild-type bacteria and a ΔsopEΔsopE2 mutant and showed an effect on RHOG membrane ruffling (5), though their work on invasion was only performed with a wild-type (SopB+SopE+SopE2+) strain (16). In terms of why they observed this phenotype while we do not, we propose three non-mutually exclusive hypotheses:

1. Differences in measures of RHOG activity and/or invasion between the studies. While we measured % Infected Cells, Patel and Galán considered either % Membrane Ruffling Cells or % Invasion by microscopy.
2. Differences in cell lines affect reliance on RHOG. The Patel and Galán papers use Henle-407 cells, while we use HeLa cells. While Henle-407 cells are a HeLa contaminant, it is possible that this lineage experiences RHOG dependent invasion not observed at measurable levels in our HeLa cells.
3. Specific conditions in the Galán lab lead to RHOG dependence. Unfortunately, there is increasing evidence in the field that the RHOG phenotype, even when measured by methods more similar to the Patel and Galán paper and/or in Henle cells (See image below), is not reproducible (7, 8). This could be due to differences in siRNA used, lab specific clones of *Salmonella* or Henle cells, or a variety of other factors.

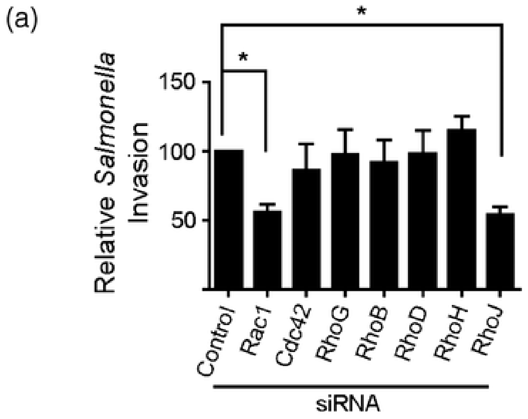

Source: Truong et al. 2018 (7)
Source Figure Legend: (a) Henle 407 cells were transfected with siRNA against the indicated Rho GTPase and infected with wild-type (WT) S. Typhimurium for 30 min. Differential antibody staining was used to distinguish between intracellular and extracellular bacteria. In three independent experiments, 200 cells were scored for bacterial internalisation. Statistical analysis was performed with one-way analysis of variance and post hoc Dunnett's test.

(3) I assume that Figure 4 experiments were conducted using HeLa cells, but I cannot find the description.

We have added "HeLa cells" to the figure title and legend.

(4) Description of Figure S1 (D) is missing in the legend.

We have corrected the figure legend.

Reviewer #3:
1. In Figure 3D, it seems that the DsopB strain is more invasive compared to the S. Typhi wild type strain Ty2 in both NT and siARHGEF26 HeLa cells. Is this difference significant, and if so, how can this be explained? It shows that SopE-dependent invasion is more efficient (when SopB is deleted), also in the absence of ARHGEF26.

This was also asked above by Reviewer 2. We have copied the response, exactly, here for convenience:

We have observed that the ∆sopB mutant typically invades equal to slightly higher than the wild-type strain, though occasionally we do see less invasion. Figures 3D (Now Figure 3B) and the experiments for Dlg1 knockdown in 4C were from the same experiments where ∆sopB did, by chance, have some of the most consistent increased invasion we've seen. As a result, it did have a significant p-value by a t-test.

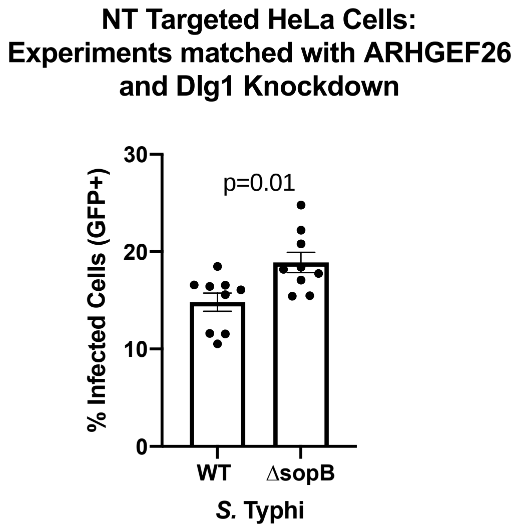

Source: This work (Derived from Figure 4)

By contrast, the experiments matched with siSCRIB knockdown did not meet significance, though there was slightly smaller N for these experiments (6 replicates per group vs 9 in the experiment above).

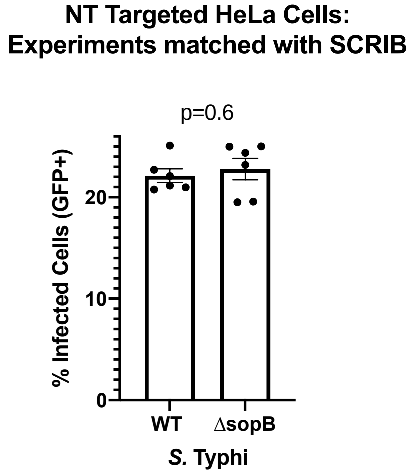

Source: This work (Derived from Figure 4)

Because the phenotype is, typically, not consistent, we do not comment on the phenotype in the study. From a biological perspective, knocking out sopB may allow for a greater amount of secretion of other effectors, such as sopE2, that may be better at stimulating invasion.

2. Would a DsopB/sopE S. Typhi mutant invade NT and siARHGEF26 cells similarly? Or are there other effectors also required for ARHGEF26-dependent invasion?

This experiment was requested and explained above. We have copied the answer here for convenience.

While it is difficult to measure *Salmonella* uptake in ∆*sopB*∆*sopE S.* Typhi by HeLa cells due to their inefficient invasion, we infected HeLa cells at a high MOI and measured ~0.5% infection, which did not vary between NT and siARHGEF26 conditions, suggesting that in HeLa cells the ARHGEF26 infection decrease is occurring by SopB/SopE-mediated mechanisms.

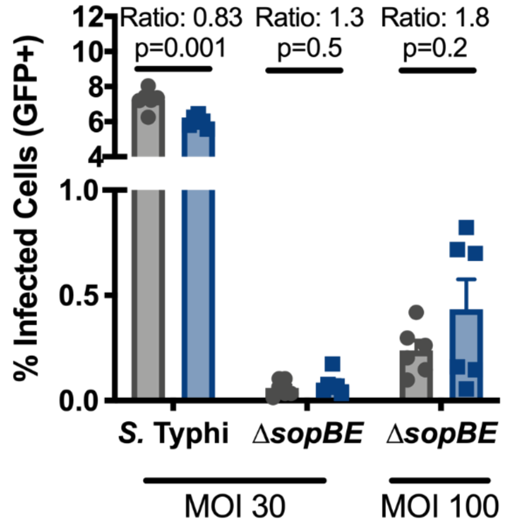

Source: This work (Derived from Figure 3)

3. Figure 6A shows that silencing of ARHGEF26 reduces IL-8 production in unstimulated cells by approximately 2-fold. SiARHGEF26 cells infected with S. Typhi of S. Tm also had reduced IL-8 production. Is this moderately reduced IL-8 production a result of the lower basal IL-8 production, or is it an effect of Salmonella-induced inflammation. Did the authors correct for the IL-8 reduction in unstimulated cells?

The magnitude of the siARHGEF26-mediated cytokine reduction for both S. Typhi and S. Typhimurium induced IL-8 was ~1,400pg/mL and ~700pg/mL, accordingly. In contrast, the absolute change in uninfected IL-8 abundance was only ~200pg/mL. Thus, while our data are not background subtracted, the differences in basal levels cannot explain our differences following stimulation. Further demonstrating this point, while we see that siARHGEF26 leads to changes in unstimulated and stimulated amounts of IL-8, we see that siRHOG *only* impacts unstimulated levels, demonstrating specificity in our system.

We have added discussion of this in the paper:

"Importantly, the magnitude of the siARHGEF26-mediated cytokine reduction for both S. Typhi and S. Typhimurium induced IL-8 was much larger than the reduction seen in uninfected cells (~1,400pg/mL and ~700pg/mL, accordingly vs ~200pg/mL. This substantially larger phenotype with infection leads us to conclude that our results are not simply consequences of lower basal levels of cytokine production following ARHGEF26 knockdown. Instead, we propose that there are multiple levels of cytokine regulation by ARHGEF26, and that this could have important implications during Salmonella infection."

Works Cited:

1. Alvarez MI, Glover LC, Luo P, Wang L, Theusch E, Oehlers SH, et al. Human genetic variation in VAC14 regulates Salmonella invasion and typhoid fever through modulation of cholesterol. Proc Natl Acad Sci U S A. 2017;114(37):E7746-E55.
2. Wang L, Pittman KJ, Barker JR, Salinas RE, Stanaway IB, Williams GD, et al. An Atlas of Genetic Variation Linking Pathogen-Induced Cellular Traits to Human Disease. Cell Host Microbe. 2018;24(2):308-23 e6.
3. Jaslow SL, Gibbs KD, Fricke WF, Wang L, Pittman KJ, Mammel MK, et al. Salmonella Activation of STAT3 Signaling by SarA Effector Promotes Intracellular Replication and Production of IL-10. Cell Rep. 2018;23(12):3525-36.
4. Gibbs KD, Washington EJ, Jaslow SL, Bourgeois JS, Foster MW, Guo R, et al. The Salmonella Secreted Effector SarA/SteE Mimics Cytokine Receptor Signaling to Activate STAT3. Cell Host Microbe. 2020;27(1):129-39 e4.
5. Patel JC, Galan JE. Differential activation and function of Rho GTPases during Salmonella-host cell interactions. J Cell Biol. 2006;175(3):453-63.
6. Awadia S, Huq F, Arnold TR, Goicoechea SM, Sun YJ, Hou T, et al. SGEF forms a complex with Scribble and Dlg1 and regulates epithelial junctions and contractility. J Cell Biol. 2019;218(8):2699-725.
7. Truong D, Boddy KC, Canadien V, Brabant D, Fairn GD, D'Costa VM, et al. Salmonella exploits host Rho GTPase signalling pathways through the phosphatase activity of SopB. Cell Microbiol. 2018;20(10):e12938.
8. Aiastui A, Pucciarelli MG, Garcia-del Portillo F. Salmonella enterica serovar typhimurium invades fibroblasts by multiple routes differing from the entry into epithelial cells. Infect Immun. 2010;78(6):2700-13.
9. Krishna Subbaiah V, Massimi P, Boon SS, Myers MP, Sharek L, Garcia-Mata R, et al. The invasive capacity of HPV transformed cells requires the hDlg-dependent enhancement of SGEF/RhoG activity. PLoS Pathog. 2012;8(2):e1002543.
10. Cloning and molecular characterization of genes whose products allow Salmonella typhimurium to penetrate tissue culture cells., (1989).
11. Hapfelmeier S, Ehrbar K, Stecher B, Barthel M, Kremer M, Hardt WD. Role of the Salmonella pathogenicity island 1 effector proteins SipA, SopB, SopE, and SopE2 in Salmonella enterica subspecies 1 serovar Typhimurium colitis in streptomycin-pretreated mice. Infect Immun. 2004;72(2):795-809.
12. Pretreatment of mice with streptomycin provides a Salmonella enterica serovar Typhimurium colitis model that allows analysis of both pathogen and host., (2003).
13. Fattinger SA, Bock D, Di Martino ML, Deuring S, Samperio Ventayol P, Ek V, et al. Salmonella Typhimurium discreet-invasion of the murine gut absorptive epithelium. PLoS Pathog. 2020;16(5):e1008503.
14. Schindelin J, Arganda-Carreras I, Frise E, Kaynig V, Longair M, Pietzsch T, et al. Fiji: an open-source platform for biological-image analysis. Nature methods. 2012;9(7):676-82.
15. Chassaing B, Srinivasan G, Delgado MA, Young AN, Gewirtz AT, Vijay-Kumar M. Fecal lipocalin 2, a sensitive and broadly dynamic non-invasive biomarker for intestinal inflammation. PLoS One. 2012;7(9):e44328.
16. Patel JC, Galan JE. Investigating the function of Rho family GTPases during Salmonella/host cell interactions. Methods Enzymol. 2008;439:145-58.
